# The Metabolic Response of Brachypodium Roots to the Interaction with Beneficial Bacteria Is Affected by the Plant Nutritional Status

**DOI:** 10.3390/metabo11060358

**Published:** 2021-06-03

**Authors:** Martino Schillaci, Cheka Kehelpannala, Federico Martinez-Seidel, Penelope M. C. Smith, Borjana Arsova, Michelle Watt, Ute Roessner

**Affiliations:** 1 School of BioSciences, University of Melbourne, Parkville 3010, Australia; ckehelpannal@student.unimelb.edu.au (C.K.); watt.m@unimelb.edu.au (M.W.); u.roessner@unimelb.edu.au (U.R.); 2 Max-Planck-Institute of Molecular Plant Physiology, Am Mühlenberg 1, 14476 Potsdam-Golm, Germany; MSeidel@mpimp-golm.mpg.de; 3 Department of Animal, Plant, and Soil Sciences, School of Life Sciences, La Trobe University, Bundoora 3086, Australia; p.smith3@latrobe.edu.au; 4 Institute for Bio & Geosciences, Plant Sciences (IBG-2), Forschungszentrum Juelich GmbH, 52425 Juelich, Germany; b.arsova@fz-juelich.de

**Keywords:** plant growth promoting (PGP) bacteria, cereals, metabolomics, lipidomics, root metabolism, *Brachypodium distachyon* Bd21-3, *Azospirillum brasilense* Sp245

## Abstract

The potential of plant growth promoting (PGP) bacteria in improving the performance of plants in suboptimal environments is increasingly acknowledged, but little information is available on the mechanisms underlying this interaction, particularly when plants are subjected to a combination of stresses. In this study, we investigated the effects of the inoculation with the PGP bacteria *Azospirillum brasilense* (Azospirillum) on the metabolism of the model cereal *Brachypodium distachyon* (Brachypodium) grown at low temperatures and supplied with insufficient phosphorus. Investigating polar metabolite and lipid fluctuations during early plant development, we found that the bacteria initially elicited a defense response in Brachypodium roots, while at later stages Azospirillum reduced the stress caused by phosphorus deficiency and improved root development of inoculated plants, particularly by stimulating the growth of branch roots. We propose that the interaction of the plant with Azospirillum was influenced by its nutritional status: bacteria were sensed as pathogens while plants were still phosphorus sufficient, but the interaction became increasingly beneficial for the plants as their phosphorus levels decreased. Our results provide new insights on the dynamics of the cereal-PGP bacteria interaction, and contribute to our understanding of the role of beneficial microorganisms in the growth of cereal crops in suboptimal environments.

## 1. Introduction

Low temperature and P deficiency are two of the most common abiotic stresses for crops, and their combination can be particularly detrimental during the early stages of the development of winter cereals in P-poor regions of the world [1].

Low temperature affects virtually all metabolic processes of plants by changing the enzyme kinetics of biochemical reactions, inhibiting photosynthesis, damaging intra- and intercellular structures, decreasing water and nutrient uptake capacity, and causing oxidative stress [2,3]. Plants use P in most of their metabolic reactions, and phosphate deficiency activates various metabolic rearrangements, which increase external inorganic P (Pi) uptake and optimize internal Pi use efficiency [4]. In the case of extended P deprivation, plants often start recycling P from P-containing biomolecules such as RNA, phospholipids and small phosphorylated metabolites, and the metabolic pathways these compounds are involved in are severely impacted by prolonged P deficiency [5].

Low temperatures can affect plant metabolic responses to low P availability and vice versa. By decreasing plant growth rate, low temperature might indirectly affect plant P requirements; furthermore, these stresses have an opposite effect on root development—which is generally stimulated by P deficiency and hindered by low temperatures [6]. Low temperature hampers photosynthesis by inhibiting sucrose synthesis, and this causes an accumulation of phosphorylated intermediates and an imbalance in the ATP/ADP ratio [7]. As mentioned before, these compounds are also affected by low P availability, as they decrease in P deficient plants. *Arabidopsis thaliana* mutants with reduced shoot Pi adapt to low temperature better than mutants with increased shoot Pi, but this might be reversed in highly P deficient plants, as these plants might not be able to readjust their metabolism to face low temperature [8]. When comparing the metabolic response of maize leaves to low temperatures and P limitation, Schlüter et al. [9] reported that plants subjected to low temperatures increased their carbohydrate and phosphorylated metabolite levels, while the opposite was observed in P deficient plants.

Plant growth promoting (PGP) bacteria can improve plant adaptation to abiotic stresses by affecting their metabolism, particularly by producing PGP compounds, improving nutrient uptake and interfering with plant stress signaling pathways [10,11,12]. The metabolic response of cereal roots to inoculation with PGP bacteria has been increasingly investigated in recent years [13,14,15,16,17], and few of those studies have analyzed the role of PGP bacteria in shaping the root metabolome of cereals facing stress [18,19]. Gagné-Bourque et al. [18] studied the impact of PGP bacteria at various stages of the interaction with timothy grass (*Phleum pratense*) subjected to drought. Their results are of particular interest as inoculation affected plant phenotype and metabolome differently through the experiment, showing the plasticity of the plant–bacteria interaction in different conditions. There is instead a lack of time-resolved studies on the effects of the interaction with PGP bacteria on the metabolome of cereal roots subjected to low P and cold temperatures, applied separately or simultaneously.

This study is part of a project that investigated the effect of the interaction with the PGP bacteria *Azospirillum brasilense* (Azospirillum) on the development of the model cereal *Brachypodium distachyon* (Brachypodium) subjected to suboptimal temperature and low P availability. In our previous phenotypic analysis, we compared the development of plants grown at suboptimal temperatures and various P levels and inoculated or not with Azospirillum. At the lowest P availability, bacteria increased shoot biomass in the early stages of the interaction, while the shoot nutrient concentration was not affected. Additionally, inoculated plants featured an increase of their branch root length in the second half of the experiment, concurrently with plant P deficiency. We hypothesized that the inoculation improved plant resistance to P deficiency stress by optimizing their root architecture for the exploration of the substrate [1]. Thus the current study focuses on investigating the metabolism of the previously phenotyped Brachypodium roots, with plants grown in the conditions where Azospirillum had a positive effect on their development [1]. We resolved the changes occurring in the root polar metabolites and lipids at various stages of the interaction with Azospirillum, and linked them to the plant nutritional status and phenotype. The levels of phosphorylated compounds decreased throughout the experiment, causing relevant rearrangements in plant metabolism. Inoculated plants synthesized defense-related compounds during the early stage of the interaction, while at later stages inoculation decreased abiotic stress. We propose that the plant P deficiency shaped the interplay with the bacteria, which in turn affected the plant response to the abiotic stresses they were subjected to.

## 2. Results

*Brachypodium distachyon* Bd21-3 (Brachypodium) [20] with or without *Azospirillum brasilense* Sp245 (Azospirillum) [21] inoculation were grown in a hydroponic system for 21 d at suboptimal temperatures (20/10 °C day/night), and throughout the experiment plants were supplied with a low P (7 µM KH_2_PO_4_) nutrient solution. At 7, 14, and 21 days after the inoculation (DAI), roots were harvested and polar metabolites and lipids were extracted and analyzed via untargeted GC-MS or LC-MS. The root metabolic profiles were compared between inoculated and non-inoculated plants at each time point, and between different time points of the same treatment.

The analysis of the plant phenotype at various time points in the interaction with Azospirillum has been reported by Schillaci et al. [1].

### 2.1. Polar Metabolites

#### 2.1.1. Root Polar Metabolites

A total of 92 polar metabolites were detected in the roots of Brachypodium, mainly belonging to the classes of sugars (25), amino acids/amines (30) and organic acids (32) (Table S1). β-gentibiose, cystine, maltotriose, melibiose, and myo-inositol-2-phosphate were present only at 7 DAI.

#### 2.1.2. P-Containing Compounds

**Figure 1:**
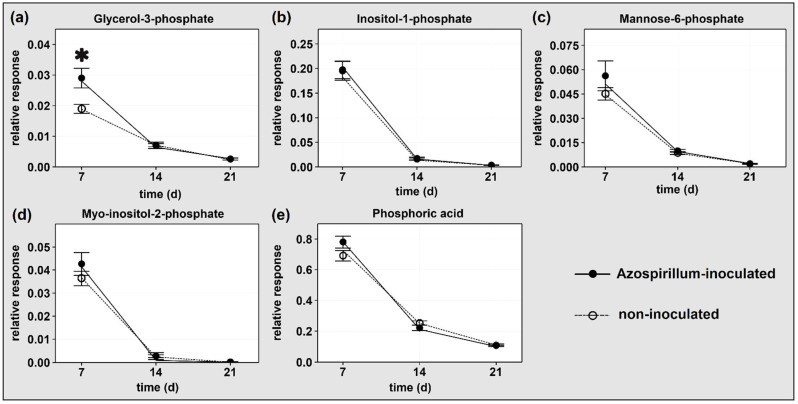
Relative response of P-containing compounds in roots of plants with or without Azospirillum inoculation and harvested after 7, 14, and 21 DAI. Means ± standard error are presented. *n* = 6 for all samples except for non-inoculated at 21 DAI (*n* = 8). Asterisks in the graphs indicate a fold change (FC) <−1.5 or >1.5 and probability of significant difference between relative response of Azospirillum-inoculated and non-inoculated plants based on Student’s *t*-test *p* < 0.05 (see also Table S1).

Five P-containing compounds were detected in the root samples: glycerol-3-phosphate (G3P), inositol-1-phosphate, mannose-6-phosphate, myo-inositol-2-phosphate, and phosphoric acid. The levels of all these compounds decreased between 7 and 21 DAI, with a similar magnitude in both treatments (Figure 1). The only compound significantly affected by the inoculation was G3P, which was more abundant (fold change (FC) = 1.53) in Azospirillum-inoculated plants at 7 DAI (Figure 1a).

#### 2.1.3. Comparison between Polar Metabolites of Inoculated and Non-Inoculated Plants

The response of polar compounds detected in Brachypodium roots was assessed using relative quantification and compared between inoculated and non-inoculated samples at each time point. Hierarchical clustering paired with a heatmap of metabolite abundances and principal component analysis (PCA) allowed the behavior of root metabolic profiles to be visualized throughout the experiment.

**Figure 2:**
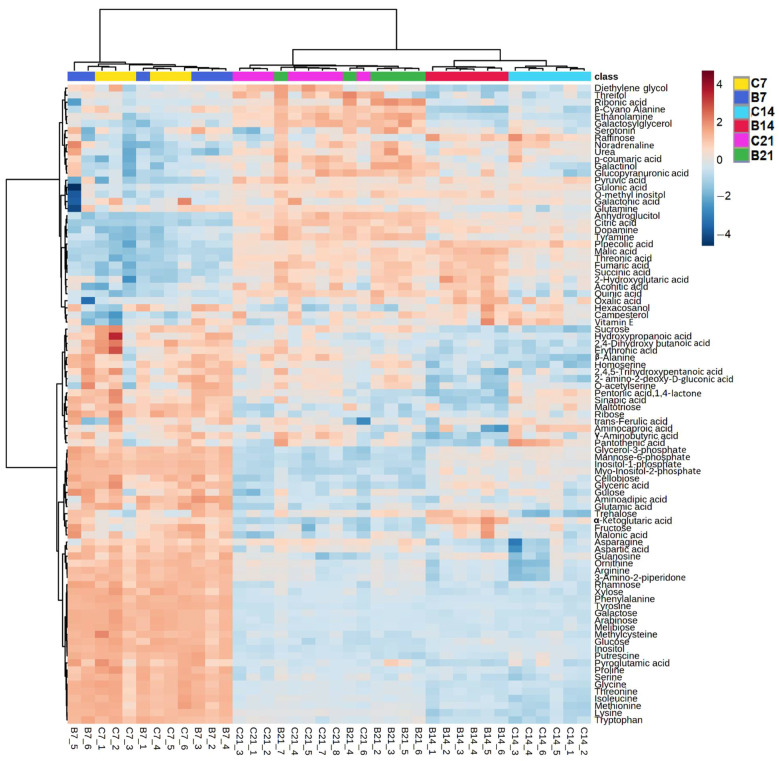
Hierarchical clustering coupled with heatmap of the polar metabolite profiles of Azospirillum-inoculated (B) and non-inoculated roots (C) of Brachypodium harvested at 7, 14, and 21 DAI. The lettering at the bottom of the heatmap indicates the replicates: for each replicate, blue and red colors indicate the lower or higher abundance of specific metabolites respectively compared to the other replicates, with darker colors indicating more pronounced differences.

Polar metabolite patterns firstly clustered based on time point, while the bacterial inoculation was a weaker discriminant (Figure 2, Figure S1). Based on the levels of detected metabolites, samples from different treatments were often more similar than samples belonging to the same treatment. The clearest separation was observed at 14 DAI, when samples from the two treatments clustered separately (Figure 2).

While most metabolites did not show significant fold changes between the two treatments at any time point, the response of a small group of metabolites varied dynamically throughout the time series (FC < −1.5 or FC > 1.5, *p* < 0.05, Table S1, Figure S2). A total of 18 compounds were significantly increased or decreased when the two treatments were compared at each time point. These included aminocaproic acid, campesterol, β-cyano-alanine, diethylene glycol, G3P, hexacosanol, malic acid, α-ketoglutaric acid (α-KG), pantothenic acid, pentonic acid, 1,4-lactone, putrescine, pyroglutamic acid, quinic acid, ribonic acid, serotonin, sinapic acid, trehalose, and xylose.

At 7 DAI, the compounds that increased in inoculated plants compared to non-inoculated were hexacosanol (FC= 2.36), campesterol (FC = 1.94), trehalose (FC = 1.58), and G3P (FC = 1.53). The only compound that decreased was aminocaproic acid (FC = −1.92) (Table S1, Figure S2a).

The highest number of changed polar metabolites was observed in root samples harvested 14 DAI. At this time point, four metabolites increased in inoculated samples compared to controls: trehalose (FC = 2.16), quinic acid (FC = 2.12), α-KG (FC = 1.99), and malic acid (FC = 1.76). Seven metabolites were reduced: pentonic acid, 1,4-lactone (FC = −2.98), aminocaproic acid (FC = −2.63), sinapic acid (FC = −2.54), β-cyano-alanine (FC = −1.95), pantothenic acid (FC = −1.87), putrescine (FC= −1.55), and diethylene glycol (FC = −1.50) (Table S1, Figure S2b).

At 21 DAI, the polar metabolites with higher abundance due to inoculation were ribonic acid (FC = 5.43), xylose (FC = 1.8), pyroglutamic acid (FC = 1.75), and serotonin (FC = 1.50). Pentonic acid, 1,4-lactone (FC = −1.73) was the only compound with reduced abundance (Table S1, Figure S2c).

Apart from pentonic acid, 1,4-lactone, trehalose and aminocaproic acid, no compounds were significantly affected at more than one time point.

### 2.2. Lipids

#### 2.2.1. Root Lipids

A total of 6294 features were detected in Brachypodium root extracts, and of those 257 were annotated as lipids. Identified species belonged to glycerolipids (GL, 178), glycerophospholipids (GP, 62), and sphingolipids (SP, 17). GLs were divided into acyl diacylglyceryl glucuronides (ADGGA, 14), diacylglycerols (DG, 27), digalactosyldiacylglycerols (DGDG, 20), diacylglyceryl glucuronides (DGGA, 11), monogalactosyldiacylglycerols (MGDG, 7), and triacylglycerols (TG, 99). GPs were divided into lysophophatidylcholines (LPC, 8), phophatidylcholines (PC, 27), phosphatidylethanolamines (PE, 16), phosphatidylglycerols (PG, 6), and phosphatidylinositols (PI, 5). SPs were divided into ceramide alpha-hydroxy fatty acid-phytospingosines (Cer-AP, 9), hexosylceramides (HexCer, 2), hexosylceramide alpha-hydroxy fatty acid-phytospingosines (HexCer-AP, 4), and ceramide phosphoinositols (Pi-Cer, 2). For the complete description of detected features, see Table S2a.

#### 2.2.2. Root Glycerophospholipids and Galactolipids

The comparison of specific lipid classes at different time points showed converse trends between GPs (PCs and PEs) and galactolipids (MGDGs and DGDGs) in both treatments (Table S2b). Of the 27 detected PCs, 13 decreased and 5 increased in inoculated plants between 7 and 21 DAI. A similar result was observed in non-inoculated plants, where 15 PCs decreased and 5 PCs increased during the same time window. The 16 detected PEs show a similar behavior, with 7 decreased and 2 increased lipid species in inoculated plants, and 7 decreased and 1 increased lipid in non-inoculated plants. Between 7 and 21 DAI, all 20 detected DGDGs increased in both treatments, while out of the 7 detected MGDGs, 5 and 4 increased in inoculated and non-inoculated plants, respectively. None of the detected galactolipids decreased during the experiment, in either of the treatments.

#### 2.2.3. Comparison between Lipids of Inoculated and Non-Inoculated Plants

**Figure 3:**
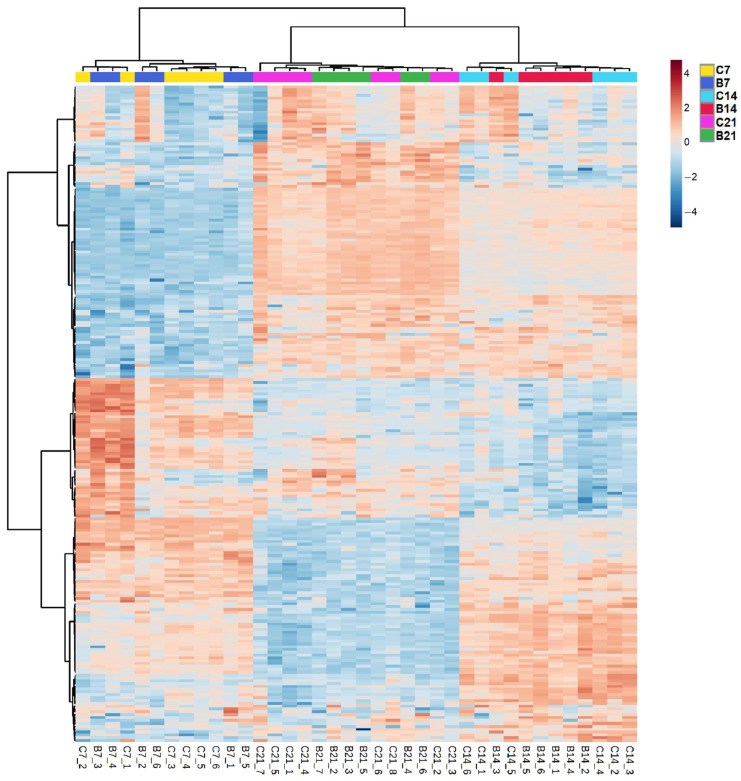
Hierarchical clustering coupled with heatmap of the lipid profiles of Azospirillum-inoculated (B) and non-inoculated roots (C) of Brachypodium harvested at 7, 14, and 21 DAI. The lettering at the bottom of the heatmap indicates the replicates: for each replicate, blue and red colors indicate the lower or higher abundance of specific lipids compared to the other replicates, with darker colors indicating more pronounced differences.

Lipid and unidentified feature profiles of inoculated and non-inoculated plants were compared at 7, 14, and 21 DAI. The hierarchical clustering and PCA plot of lipid species and unidentified features detected at different time points show that features separated based on time of harvest. Lipid species did not separate based on the bacterial treatment (Figure 3, Figure S3), while a more pronounced clustering was observed in unidentified features, particularly at 7 and 21 DAI (Figure S4).

The analysis of lipid species in the two treatments at different time points shows a limited effect of the inoculation on individual features. DGDG 36:1, LPC 14:0, and PE 33:2 were less abundant in inoculated plants at 7 DAI; PC 30:1 was more abundant in inoculated plants at 14 DAI. No lipid species differed significantly at 21 DAI (Figure S5).

**Figure 4:**
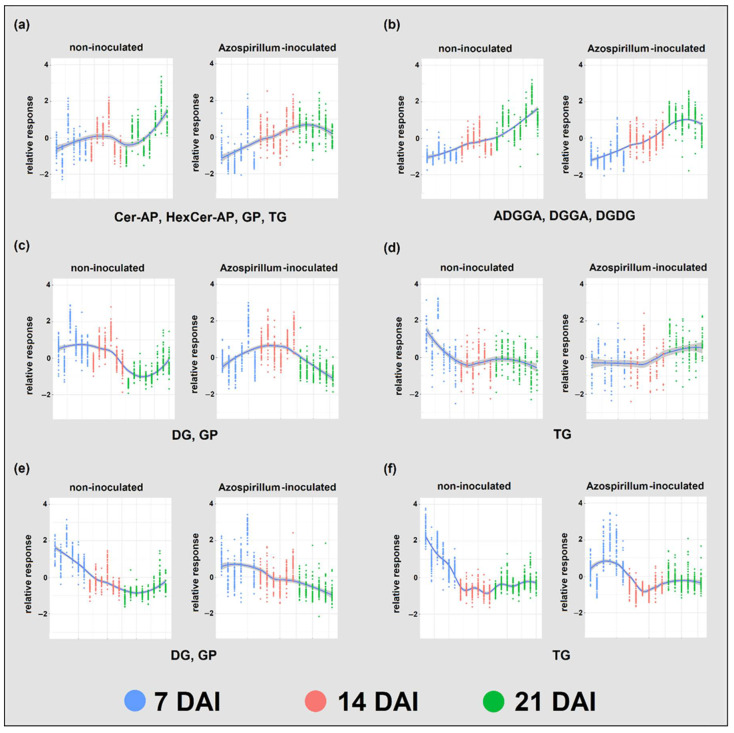
Profile of lipid species identified in Azospirillum-inoculated and non-inoculated samples harvested at 7, 14, and 21 DAI using K-means clustering. Clusters formed in the two treatments were paired based on their lipid species composition (**a**–**f**). At each time point, biological replicates are displayed as lines of dots, where each dot represents a lipid species. The main lipid classes of each cluster couple are also displayed. Shade of the fitting line represents the standard deviation within metabolite groups. ADGGA: acyl diacylglyceryl glucuronide, Cer-AP: ceramide alpha-hydroxy fatty acid-phytospingosine, DG: diacylglycerol, DGDG: digalactosyldiacylglycerol, DGGA: diacylglyceryl glucuronide, GP: glycerophospholipids, HexCer-AP: hexosylceramide alpha-hydroxy fatty acid-phytospingosine, TG: triacylglycerol.

To further investigate the effects of bacterial inoculation on the Brachypodium root lipidome, K-means clustering analysis was performed separately on the lipids identified in inoculated and non-inoculated samples, which were grouped based on their levels at 7, 14, and 21 DAI (Figure 4). Seven lipid clusters were fitted and grouped between the two treatments based on their metabolite composition. The degree of similarity between the clusters in each pair was generally good, varying between 48.7 and 75.5% for six out of the seven paired clusters (for the complete cluster composition and the similarity scores of each cluster pair, see Table S2c). The clusters of inoculated and non-inoculated samples differed particularly in the third and fourth pairs. In the third pair (Figure 4c), DGs and GPs identified in non-inoculated samples were generally stable between 7 and 14 DAI, and decreased at 21 DAI, while those from inoculated samples increased between 7 and 14 DAI and decreased again between 14 and 21 DAI. In the fourth pair (Figure 4d), TGs from non-inoculated samples decreased between 7 and 14 DAI and were generally stable between 14 and 21 DAI, while features from inoculated samples were stable between 7 and 14 DAI and increased between 14 and 21 DAI.

## 3. Discussion

The comparison of polar metabolites and lipids extracted from the roots of Azospirillum-inoculated and non-inoculated roots of Brachypodium at 7, 14, and 21 DAI revealed that the time of the harvest was a strong discriminant between samples, while the bacterial treatment affected specific sections of root metabolism at different time points.

### 3.1. Polar Metabolites

#### 3.1.1. Plants Consumed Phosphorylated Compounds to Face the Increasing P Deficiency

All detected P-containing compounds decreased in both treatments through the experiment (Figure 1) as might be expected given the progressive decrease of shoot P concentration in those same plants [1]. The reduction in phosphoric acid indicates that the inorganic P reserves in the vacuole were declining (Figure 1e), a process associated with the reduction in phosphorylated metabolites as internal P recycling from senescing tissues occurred [22]. Plants progressively switched to metabolic pathways that require little or no phosphorylated compounds, allowing P to be remobilized for essential metabolism [5]. The large reduction in all detected P-containing compounds suggests that not only were the shoots of both treatments increasingly P deficient [1] but this was also the case in the roots. As previously reported, despite a similar metabolic response indicating P deficiency the growth of shoots and roots was stronger in plants inoculated with Azospirillum.

#### 3.1.2. Azospirillum Elicited Different Responses in Plants at Different Stages

The profile of polar metabolites extracted from Brachypodium roots with and without Azospirillum inoculation changed through time, suggesting that bacterial interactions dynamically and specifically altered plant metabolism at low temperature and P conditions. The time point at which inoculation induced the largest number of metabolite changes was 14 DAI. Eleven metabolites had significant changes, of which 4 were increased and 7 were decreased by inoculation. Changes in polar metabolites were more limited at 7 and 21 DAI, with four compounds increased and one decreased in inoculated plants at both time points (Table S1, Figure S2).

**Figure 5:**
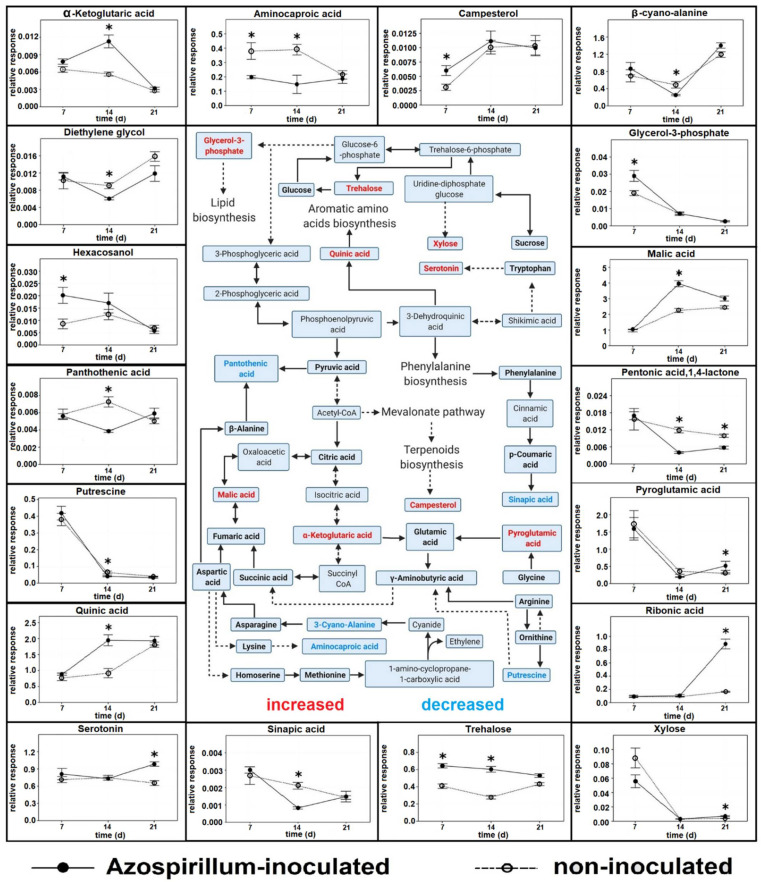
Mapping of the polar metabolites affected by inoculation with Azospirillum at 7, 14, or 21 DAI in relation to primary metabolism pathways. In the central diagram, detected compounds appear in bold font, and a dotted line connecting two compounds indicates that further intermediates are present. In the surrounding plots, asterisks in the graphs indicate a FC < −1.5 or >1.5 and probability of significant difference between mean of Azospirillum-inoculated and non-inoculated plants based on Student’s *t*-test *p* < 0.05.

The metabolic pathways associated with significant changes in this study are shown in Figure 5. Compounds significantly affected by the inoculation are scattered across primary metabolism rather than belonging to specific pathways, with the partial exception of sugar metabolism (trehalose, xylose) and the citric acid cycle (malic acid, α-ketoglutaric acid(α-KG)). It was not possible to map hexacosanol, diethylene glycol, pentonic acid, 1,4-lactone, and ribonic acid onto Figure 5, because a pathway for those compounds has not been characterized in plants yet. Further information on affected compounds in relation to stresses and/or interaction with PGP bacteria can be found in Appendix A Table A1.

Dynamic metabolite fluctuations in association with bacterial inoculation are evident by mapping affected compounds at different time points (Figure 5). This reveals metabolites which were most significantly changed in response to growing conditions (time, inoculation).

We cannot exclude that compounds increased in inoculated plant roots were produced by the bacteria or by both bacteria and the plant but, due to the great disproportion between host plant and bacterial biomass, it is unlikely that bacterial metabolites had a significant impact on the observed results.

It is noteworthy that all compounds whose levels were increased in inoculated plants at 7 DAI—hexacosanol, campesterol, trehalose, and G3P—can be involved in plant response to biotic stresses and this suggests that the plant was initially producing antimicrobial compounds to limit bacterial colonization of the roots. Hexacosanol stimulates wax barriers formation, and can also be one component of the wax layer suberin, a structure involved in plant response against biotic and abiotic stresses [23,24]. Campesterol is a precursor for the synthesis of brassinosteroids, a class of hormones involved in plant responses to environmental stresses, both biotic and abiotic [25,26,27]. Brusamarello-Santos et al. [14] report that trehalose levels increased in maize inoculated with *Azospirillum brasilense* or *Herbaspirillum seropedicae*, and the authors hypothesize a role of this compound in the regulation of the plant—bacteria interaction, possibly in defense related mechanisms. G3P plays an essential role as a signaling molecule in plant systemic acquired resistance, a defensive mechanism against a broad variety of pathogens which is activated during early infection stages, preventing pathogen propagation in distal tissues [28,29]. This type of defense response is not unusual; PGP bacteria can be sensed as pathogens during early stages of the interaction, as both beneficial and detrimental microorganisms can trigger the plant immune system [17]. Once different recognition mechanisms of plant and microorganisms are established, the plant response to the PGP bacteria changes as was seen at 14 and 21 DAI.

Brassinosteroids, the phytohormones synthesized from campesterol, are normally involved in cell elongation [30], and specific concentrations can stimulate branch root formation [31]. The untargeted analysis we performed only allowed us to compare the levels of detected compounds and did not provide information on their absolute quantity, but it is possible that the higher campesterol levels in inoculated Brachypodium at 7 DAI (Figure 5, Table S1) caused the earlier development of branch roots observed in those plants [1]. As branch roots are of great importance for plants growing in a P-poor environment [32] this could have produced a significant advantage for Azospirillum-treated plants.

Trehalose and sucrose are sugars separated by two enzymatic reactions only (Figure 5) and can accumulate in plants subjected to cold stress [33]. Compared to sucrose, trehalose biosynthesis requires much smaller amounts of carbon, and allows the uridine diphosphate glucose and glycerol-6-phosphate pools to be conserved [34]. In stressed plants, the metabolism of sucrose and trehalose is typically related [33], but in our study the two compounds had analogous trends only in non-inoculated plants, with a decrease during the second week of the experiment and an increase during the third. In contrast, the trehalose content was relatively stable in inoculated plants (Figure 5), while the sucrose content followed the same trend as in non-inoculated plants (Figure S6). This suggests that the bacteria caused a shift in sugar metabolism of inoculated plants, redirecting it towards less P-consuming pathways. Finally, genes involved in trehalose biosynthesis differed in the roots of two rice cultivars subjected to various stresses, including P deficiency [35]. Plants with higher expression of such genes had shorter primary root and better developed shoots when subjected to P deprivation, which is consistent with the phenotype of Brachypodium plants inoculated with Azospirillum [1]. We hypothesize a role for trehalose in shaping the development in cereals facing P deficiency, in particular preventing the redirection of resources from the shoot to the root growth, which is a common response of plants to this stress [36].

Compared to samples harvested at 7 DAI, those from 14 DAI show a higher number of affected metabolites (Figure 5, Table S1). The characteristics of most compounds whose levels were changed at 14 DAI—quinic acid [37], α-KG [38], malic acid [5,39,40], β-cyano-alanine [41,42], and sinapic acid [43]—suggest inoculation with Azospirillum provided protection against both nutrient deficiency and low temperature stress (see also Appendix A Table A1). This hypothesis is supported by the reduction in compounds with antimicrobial activity compared 7 DAI in inoculated plants, as only sinapic acid is reported to be involved in plant defense mechanisms against pathogens [44].

Since plants undergoing P deficiency exudate organic acids to increase substrate P availability [39], they need to use amino acids as alternative carbon sources for various metabolic processes. One of the by-products of amino acid degradation is NH_4_^+^, which can reach toxic concentrations in the plant. To reduce NH_4_^+^ levels, plants can use amination of the organic acid α-KG to synthesize specific amino acids and amines (asparagine, glutamine, putrescine). This leads to reduced abundance of α-KG in plants facing severe P starvation [5] as was seen in non-inoculated plants between 7 and 14 DAI in our study (Figure 5). In inoculated plants the abundance of α-KG increased at 14 DAI. As the production of this organic acid does not seem to be part of the *A. brasilense* strategy to increase P availability [45], this result suggests that the bacterial interaction with Brachypodium preserved the plant TCA cycle, preventing the use of α-KG in reducing ammonium levels at this stage. This hypothesis is also supported by the fact that at 14 DAI, inoculated plants had lower levels of putrescine (Table S1, Figure 5), one of the compounds used by plants to incorporate NH_4_^+^.

The higher levels of β-cyano-alanine in non-inoculated Brachypodium roots suggests that they were facing higher levels of stress compared to inoculated plants. β-cyano-alanine is a product of the degradation of cyanide which, in turn, is the biproduct of the conversion of 1-amino-cyclopropane-1-carboxylic acid (ACC) to ethylene, a hormone whose synthesis is enhanced in plants facing stress [42]. Thus, stressed plants can often produce high levels of cyanide, which needs to be quickly metabolized as it inhibits electron transport and metalloenzymes [46]. The main pathway for this process is the β-cyano-alanine synthase pathway, which incorporates the cyanide into a cysteine molecule forming β-cyano-alanine, subsequently converted into asparagine, aspartate, and ammonia, and these compounds can be used for plant primary metabolism [47]. In this study, inoculated Brachypodium plants had significantly lower β-cyano-alanine content at 14 DAI (Table S1, Figure 5), which suggests a lower production of ethylene at this specific time point and hence lower stress levels. Non-inoculated plants had higher levels of asparagine (FC = 2.63) compared to the inoculated ones. Unfortunately, asparagine quantification is often problematic using GC-MS and LC-MS is preferred [48]. The detected levels in our samples varied substantially within each treatment resulting in asparagine FC at 14 DAI being not statistically significant (*p*-value = 0.68, see Table S1). However, this result at least partially supports the hypothesis of cyanide detoxification occurring in non-inoculated plants, as asparagine is one of the products of the β-cyano-alanine synthase pathway (Figure 5).

A smaller number of metabolites differed significantly between the two treatments at 21 DAI, and the identity of most compounds changed compared to the previous time point (Table S1, Figure 5). This suggests a change in the plants’ strategy to face the increasing P deficiency in their tissues. As at 14 DAI, some affected compounds—ribonic acid [49] and serotonin [50]—may play a role in plant adaptation to suboptimal conditions, rather than limiting bacterial growth as observed at 7 DAI.

Ribonic acid levels were generally steady through time in non-inoculated plants, while there was a great increase in inoculated plants between 14 and 21 DAI (Figure 5). In a recent study, the foliar application of γ-aminobutyric acid (GABA) increased the leaf concentration of ribonic acid in the leaves of the grass *Agrostis stolonifera* subjected to drought and improved their adaptation to this stress [51]. Azospirillum has been reported to increase the GABA concentration in the tissues of inoculated sorghum plants [52], and similar results were found in our study, where the levels of GABA increased in inoculated plants between 14 and 21 DAI (Figure S7). This suggests a connection between ribonic acid and GABA in Brachypodium’s responses to abiotic stresses.

Serotonin, also known as 5-hydroxytryptamine, is an indoleamine mainly synthesized in plant roots [53] and various studies suggest that serotonin can play a primary role in plant resistance against biotic [54] and abiotic [50] stresses. Most importantly, serotonin can shape root architecture of plants, modulating the development of different root types. Arabidopsis primary and lateral root growth is inhibited by high exogenous levels of serotonin, which affects cell division and elongation, particularly of the stem cell niche. Conversely, lower serotonin levels, while not affecting primary root, increased root branching by stimulating the maturation of lateral root primordia [55,56]. The difference in serotonin levels of the two treatments at 21 DAI (Table S1, Figure 5) could suggest a role of serotonin in shaping the root architecture of Brachypodium, which differed in inoculated plants particularly in the last week of the experiment [1]. Our study did not differentiate between the metabolomes of primary and branch roots, and future studies should determine whether serotonin concentrations differ between inoculated root types, possibly inhibiting primary root growth while at the same time stimulating branch roots.

### 3.2. Lipids

Untargeted analysis of lipid compounds extracted from Azospirillum-inoculated and non-inoculated Brachypodium roots at various time points of their growth provided three major insights. First, lipids changed in roots grown under both conditions similarly through time, thus indicating a developmental effect on root lipid composition. Secondly, both treatments reacted in a similar way to the increasing P deficiency, with a strong increase in galactolipids and a strong decrease in GP levels. Finally, the bacterial treatment did not significantly affect the root lipid profile, at any of the time points; however, specific lipid classes behaved differently through time in the two treatments.

#### 3.2.1. P Deficiency Strongly Remodeled Root Lipid Profiles of Both Treatments

The levels of both GPs and galactolipids fluctuated similarly in both treatments, with most GPs decreasing and most galactolipids increasing between 7 and 21 DAI (Table S2b). As mentioned previously, plants subjected to P deficiency can react by recycling internal P for essential metabolic processes. GPs are second only to RNA as the most abundant phosphorylated compounds in plants, and their replacement with non-phosphorous lipids in plasma membranes, mitochondrial membranes, and tonoplasts is an established strategy in roots of various cereal crops to deal with P deficiency [57].

In particular, GPs can be converted to MGDGs and DGDGs, and the latter can then form a bilayer with similar characteristics to the GP bilayer in cell membranes [58,59]. When oat plants were supplied with varying P levels for 30 days, low P caused an increase in the DGDG/PC ratio in plant roots through time, while this ratio remained stable in plants supplied with optimal P levels [57]. As our study did not include a P-sufficient treatment, we cannot exclude that the decrease of GPs and concurrent increase of galactolipids in both the treatments through time was caused by the aging of the plant. Nevertheless these trends, together with the decreased shoot P concentration [1] and the reduction in P-containing polar compounds (Figure 1) observed in both treatments, are further indications of the increasing P deficiency that plants were facing.

#### 3.2.2. Azospirillum Inoculation Had a Limited Effect on Brachypodium’s Root Lipids

The comparison of K-means clusters of identified features through time revealed that when treated as a single class of mean responses the GP, DG, and TG lipid classes behaved differently in the two treatments through time (Figure 4).

Conversely, the comparison of root lipid profiles and of the abundances of single lipid species between the two treatments revealed a weak effect of the bacterial inoculation. No separation between the Azospirillum-inoculated and non-inoculated samples was observed at any time point (Figure 3) and lipids significantly affected by the inoculation during the experiment were limited: three lipid species were less abundant in inoculated plants at 7 DAI and one was more abundant in inoculated plants at 14 DAI (Figure S5). Inoculation effects were more profound on unidentified features, which displayed a clearer clustering at different time points (Figure S4).

Very few studies have so far focused on the effect of beneficial bacteria on the root lipid composition of plants, either grown in optimal conditions or facing stresses. Due to the importance of GPs in cell membranes, most studies focus on the effects of bacterial inoculation on this lipid class. The K-means clustering suggests that, through time, GPs, might have behaved differently in the two treatments (Figure 4c). As DGs can be a product of GP hydrolysis, the fact that their levels changed similarly through time is noteworthy, but further studies on the importance of this lipid class in interactions between plants and PGP bacteria are required.

Zhang et al. [60] report that in the interaction between soybean and rhizobia, TG synthesis was decreased in nodules compared to roots. Conversely, the halophyte *Bassia indica* grown under salt stress showed improved growth and increased TG content in shoots when inoculated with the PGP bacteria *Bacillus subtilis* [61]. Calderon-Vazquez et al. [62] hypothesize that TG degradation could be part of the maize response to P deficiency, to provide acyl groups for the synthesis of glycolipi ds (e.g., galactolipids) as GPs substitutes. Overall, the available studies on the link between plant TGs, PGP bacteria, and P deficiency are still scarce and results are inconsistent, but the different behavior of this class of lipids between inoculated and non-inoculated plants in our study (Figure 4d) suggests that they may be involved in plant response to inoculation and could have been involved in plant adaptation to low P.

Although in the earlier stages of the experiment (7 DAI) a number of defense-related polar metabolites were affected, the same was not observed for the identified lipid species, despite membrane lipids being pivotal in plant interactions with microorganisms [63]. In general, the limited effect of inoculation on Brachypodium root lipid profile could have different explanations. Most of the cited studies on plant–microbe interactions showed lipid rearrangements in the first stages of the interaction (12–48 h) [64,65]. It is possible that, while the immune response involving polar metabolites continued at least until 7 DAI, the lipid response happened mainly in the first hours/days of the interaction. Since many of the membrane lipids involved in plant response to biotic interactions are GPs, it is also possible that Brachypodium response to Azospirillum was heavily affected by the increasing P deficiency they suffered through the experiment, and this would also explain why the number of lipids significantly affected by the inoculation gradually decreased during the experiment. The third possible explanation is that some affected lipids lie among the features that were not identified as lipids using the currently available libraries, as reliable and comprehensive databases for the identification of detected lipid species are still under construction [66]. Finally, the fact that the levels of identified lipid species did not vary between the two treatments does not necessarily mean that plants were not synthetizing more or less of certain lipid species and those changes were hidden by the bacterial production or consumption of those same compounds. Transcriptomic analysis of inoculated and non-inoculated plant roots could help clarify this hypothesis, by linking the abundance detected metabolites with the expression of the genes involved in their synthesis and/or degradation.

Differences between inoculated and non-inoculated plants were observed in the fluctuations of specific lipid classes by K-means cluster analysis. Compared to other unsupervised methods for the graphical representations of high-dimensional data such as PCA and hierarchical clustering, K-means clustering can be more effective in identifying subtle changes in metabolite abundance [67], and allowed the effect of inoculation on DGs, GPs and TGs to be detected but this will need to be further investigated in future studies.

## 4. Materials and Methods

### 4.1. Plant Growth Conditions

The experimental setup and conditions for plant inoculation and growth are described in detail in Schillaci et al. [1]. Briefly, 48-h-old *Brachypodium distachyon* Bd21-3 (Brachypodium) seedlings were inoculated by submerging them in a bacterial solution of *Azospirillum brasilense* Sp245, while sterile PBS was used as mock-inoculum for non-inoculated seedlings. Plants were then grown for 21 d in the *GrowScreen-PaGe* platform [68], a hydroponic system where they were supplied weekly with a modified Hoagland solution containing extremely low (7 µM KH_2_PO_4_) P levels. For the whole experiment, plants were grown at 20/10 °C day/night temperature. Plants were harvested at three time points: at 7 DAI (84 inoculated and 87 non-inoculated); at 14 DAI (24 inoculated and 36 non-inoculated) and at 21 DAI (25 inoculated and 34 non-inoculated). In order to minimize variations caused by circadian changes in the plant metabolism [69], all tissue harvesting was done at the same time of the day.

Roots were separated from shoots using a scalpel and immediately snap-frozen in liquid nitrogen. Frozen root tissues were ground to a fine powder using a Retsch Mixer Mill MM400 (Retsch GmbH, Haan, Germany) and plants from the same treatment and time point were pooled into samples with approximately the same number of individuals and fresh weight, measured using a digital scale with a 0.1 mg resolution (Mettler Toledo, Columbus, OH, USA). Six pools were formed for each treatment × time point, except for non-inoculated plants at 21 DAI which had eight groups. Samples were kept frozen during the grinding and pooling steps, to maintain quenched metabolism and thus prevent degradation of metabolites.

### 4.2. Polar Metabolites Extraction from Roots

For polar metabolites analysis, a variation of the method described in Cheong et al. [70] was used. 250 µL 100% LC-MS grade MeOH (Roth, Karlsruhe, Germany), containing 4% ^13^C_6_ sorbitol/valine (Sigma-Aldrich, Castle Hill, Australia) was added to approximately 25 mg of frozen root tissue from each pooled group. Tubes were shaken at 800 rpm for 15 min at 30 °C, centrifuged at 15,700× *g* for 15 min at room temperature and the supernatant collected. The pellet was re-extracted with 250 µL of Milli-Q H_2_O as above and the supernatants combined. In case of cloudy supernatant or precipitate observed during supernatant transfer, the pooled supernatant was centrifuged at 15,700× *g* for 10 min at room temperature before transferring as much supernatant as possible to a new tube. From each sample, 50 µL aliquots of the supernatant were transferred into glass insert (Agilent, Santa Clara, CA, USA) and dried under vacuum at 30 °C for 90 min.

### 4.3. Lipid Extraction from Roots

Lipids were extracted using a single step extraction protocol [71] as described by Kehelpannala et al. [72]. Briefly, 10 mg of frozen root tissue from each pooled group was homogenized with 400 µL of isopropyl alcohol (Roth, Karlsruhe, Germany) containing 25 µM deuterated cholesterol (internal standard, Sigma-Aldrich, Munich, Germany) and 0.01% butylated hydroxytoluene (BHT, Sigma-Aldrich, Munich, Germany). The tubes were then shaken at 1400 rpm for 15 min at 75 °C. Once cooled to room temperature, 1.2 mL of a mixture of chloroform: MeOH: water (30:41.5:3.5, v:v:v) mixture were added to each sample (all reagents were LC-MS grade and purchased from Roth, Karlsruhe, Germany). The tubes were then shaken at 300 rpm for 24 h at 25 °C and centrifuged at 15,700× *g* for 15 min at room temperature. The supernatant was then dried down with a N_2_ gas stream for approximately 50 min.

### 4.4. GC-MS Analysis of Polar Metabolites

Polar metabolite extracts were derivatized as described by Dias et al. [48], and 1 µL of sample was injected onto the GC column using a hot needle technique. Each sample was injected pure (splitless) and 1:30 diluted (split), in order to detect both lowly and highly abundant metabolites within the dynamic range of the instrument.

The GC-MS system used for the analysis was composed of Gerstel 2.5.2 autosampler, a 7890A Agilent gas chromatograph and a 5975C Agilent quadrupole mass spectrometer (Agilent, Santa Clara, CA, USA).

Gas chromatography was performed following the method described by Hillyer et al. [73], with the only differences that samples were run through a 30 m Agilent J & W VF-5MS column with 0.25 µm film thickness and 0.25 mm internal diameter with a 10 m Integra guard column.

The chromatograms and mass spectra obtained from the split and splitless injections were analyzed using the Agilent MassHunter Qualitative Analysis software (v B.07.00, Agilent Technologies, Santa Clara, CA, USA) and the AMDIS software (v 2.73, (National Institute of Standards and Technology, Gaithersburg, MD, USA) for identification of the compounds, and the Agilent MassHunter Quantitative Analysis software (v B.08.00) for their quantification. The commercial mass spectra library NIST (http://www.nist.gov, accessed on 16 September 2020) and the in-house Metabolomics Australia mass spectral library were used as references for the compound identification. During the compound identification step, chromatograms with high peak intensity analysis for each treatment/time point set of samples were selected to build the list of detected compounds in the samples.

The resulting data were normalized to the ^13^C_6_ sorbitol/valine internal standard value, and to the exact weight of tissue used for metabolite extraction. When integrating the data from compounds detected in both the splitless and the split samples run, data from the splitless run were preferred, unless the chromatogram showed poor quality due to the overloading of the column or the overlap of different compounds.

### 4.5. LC-MS Analysis of Lipids

Dried lipid extracts were resuspended in 200 µL of a butanol: MeOH (1:1, *v*/*v*) mixture containing 10 mM ammonium formate. Eight pooled biological quality control (PBQCs) samples were prepared by pooling 10 µL from each resuspended sample, and aliquoting this sample into eight vials.

Untargeted analysis of samples extracted from Brachypodium roots was then performed using the liquid chromatographic conditions derived from those described by Kehelpannala et al. [72], but using an injection volume of 10 µL. Lipids were analyzed using a Sciex Triple TOF^TM^ 6600 QqTOF mass spectrometer (Sciex, Framingham, MA, USA) equipped with a Turbo VTM dual-ion source (electro-spray ionization (ESI) and atmospheric pressure chemical ionization (APCI)) and an automated calibrant delivery system (CDS) using Information Dependent Acquisition method (IDA) in positive ion mode. The parameters were set as follows: MS1 mass range, 80–1500 *m/z*; MS2 mass range, 80–1000 *m/z*; time of flight (ToF) MS accumulation time, 250 ms; TOF MS/MS accumulation time, 40 ms; collision energy, +40 V; cycle time, 3579 ms. The following ESI parameters were used: source temperature, 450 °C; curtain gas, 45 psi; Gas 1, 45 psi; Gas 2, 45 psi; declustering potential, +150 V; Ion spray voltage floating, 4500 V. The instrument was calibrated automatically with the CDS delivering APCI calibration solution every five samples. A total of 46 samples (38 biological samples + 8 PBQCs) were analyzed.

Three root PBQC extract samples were further analyzed using a different IDA method and a Sequential Window Acquisition of All Theoretical Fragment-ion Spectra (SWATH) method, both in positive ion mode. For the SWATH analysis, the HPLC settings were the same as previously described, except that 15 µL of sample was injected in the column. The same mass spectrometer employed previously was set as described by Kehelpannala et al. [74].

For the new IDA method, the following MS settings were used: MS1 and MS2 mass range of 100–1700 Da, TOF MS accumulation time of 200 ms, TOF MS/MS accumulation time of 25 ms, collision energy of +45 V and period cycle time of 2750 ms. ESI parameters were: source temperature, 250 °C; curtain gas, 30 psi; Gas 1, 25 psi; Gas 2, 25 psi; declustering potential, +80 V; Ion spray voltage floating, 5000 V.

The features obtained from the IDA and SWATH analyses of the PBQC samples were annotated using the internal lipid library of MS-DIAL v4.12 (RIKEN CSRS, Yokohama City, Japan) [75] and the features were processed using the following settings: MS1 tolerance, 0.01 Da; MS2 tolerance, 0.025 Da; minimum peak height, 1000 amplitude; mass slice width, 0.1 Da; sigma window value, 0.5 and amplitude of MS/MS abundance cutoff, 0. The retention time of lipids in the MS-DIAL lipid library was not used as a parameter to identify lipids. The lipids were annotated using the following parameters: MS1 accurate mass tolerance, 0.01 Da; MS2 accurate mass tolerance, 0.05 Da; identification score cut off, 80% and adduct ion settings, [M^+^H]^+^, [M^+^NH_4_]^+^, [M^+^Na]^+^, [M^+^CH_3_OH^+^H]^+^, [M^+^K]^+^, [M^+^ACN^+^H]^+^, [M^+^H∙H_2_O]^+^, [M^+^2H∙H_2_O]^+^, [M^+^2Na∙H]^+^, [M^+^IsoProp^+^H]^+^, [M^+^CAN^+^Na]^+^, [M^+^2K∙H]^+^, and [M^+^DMSO]^+^. The peaks were aligned using a retention time tolerance of 0.05 min and MS1 tolerance of 0.015 Da. Then, a list of annotated lipids with their respective retention times was compiled.

The compiled list of lipids was used to annotate the mass spectrometric features extracted from the IDA of the root samples. The same settings described previously were used, except for the alignment parameter settings, where a retention time tolerance of 1 min and MS1 tolerance of 0.02 Da were used.

MS-DIAL output data consisting of peak areas of annotated lipids and unidentified features was preprocessed prior to comparing them among the different treatments and time points. Peak area of all detected features were normalized to the fresh weight of each sample, and features with coefficient of variation $CV=\frac{\mathrm{SD}\left(\mathrm{PBQCs}\right)}{\mathrm{mean}\left(\mathrm{PBQCs}\right)}$ above 20% were discarded, to ensure the reproducibility of the results [76].

### 4.6. Statistical Analyses and Data Visualization

The abundances of polar metabolites and lipids were compared using the web-based tool Metaboanalyst 4.0 (https://www.metaboanalyst.ca/, accessed on 13 November 2020). Lipid data were normalized by median, and both polar metabolite and lipid data were log transformed and auto scaled.

The metabolite profiles of the two treatments at each time point were displayed using 2D principal component analysis (PCA) and hierarchical cluster analysis of the root metabolome paired with a heatmap of metabolite abundances, with samples and features clustered using Euclidean distance function and Ward clustering algorithm.

The log transformed and auto scaled level of the compounds detected in the two treatments was compared at each time point, and the statistical significances between the measured abundances were analyzed using the Student’s *t*-test (group variance assessed using the Levene’s test). Compounds with a fold change (FC) < −1.5 or >1.5 and with raw *p*-value < 0.05 were considered differently abundant between inoculated and non-inoculated treatments.

K-means clustering of the relative responses from identified lipid species through time was performed in RStudio v. 1.2.5019 (Rstudio, Boston, MA, USA) [77]. A function named “KmeansPlus” was written and compiled in the publicly available R package RandoDiStats. The built function uses and depends on the R package ComplexHeatmap [78] as is detailed in the GitHub repository (https://github.com/MSeidelFed/RandodiStats_package, accessed on 22 December 2020). The function call defaults were used. Briefly, Pearson correlation was taken as a similarity distance between lipid variables, average was taken as the cluster construction method and a clustering consensus from 1000 runs was compiled to prevent misrepresentation from an outlier run. Finally, seven partitions or clusters were formed based on the encountered patterns between conditions. The R function returned in the environment a data frame containing the lipid variables that belong to each of the produced clusters, easing the interpretation of results. Additionally, in the working directory, plots dependent on ggplot2 [79] were generated. The plots featured the mean intensity of lipids that belong to each cluster as solid-colored lines and the standard error as shade around the mean.

## 5. Conclusions

In summary, the comparison between the root metabolic profiles of inoculated and non-inoculated plants at different stages of their growth tells us that the interaction with Azospirillum affected Brachypodium metabolism dynamically through time, particularly with regard to polar metabolites.

During the early stages of the experiment, we hypothesize that bacteria were sensed as pathogens by the host, and Brachypodium produced polar compounds with antimicrobial properties in order to limit the bacterial colonization of roots. This trend changed at later stages of the experiment, when fewer and different defense compounds were produced, and most affected compounds suggest an improved response to P deficiency by inoculated plants. This hypothesis is supported by their enhanced root development compared to non-inoculated plants during the second half of the experiment [1].

We cannot exclude that some of the compounds detected in inoculated plants might be synthesized by Azospirillum when interacting with Brachypodium, and further research is required to determine with certainty which organism produced those metabolites. In order to resolve the mechanisms for promotion of Brachypodium plant growth by Azospirillum when plants were subjected to simultaneous abiotic stresses, our experimental setup was highly controlled and simplified. However this setup allowed detailed phenotypic analysis that would have been almost impossible in a soil based system. This study lays the foundation for future studies where cereals and bacteria interact in conditions closer to an agricultural setting, such as soil substrates characterized by fluctuating humidity and temperatures, and by the native microbiota. Further studies of the interaction between Brachypodium and Azospirillum in hydroponic systems will allow the accurate characterization of the root exudates, an essential component of plant–bacteria association [80,81].

In conclusion, we suggest that the increasing P deficiency changed the interaction between Brachypodium and Azospirillum, as the beneficial effects of bacterial inoculation were most evident at 14 and 21 DAI, and at those time points inoculated roots did not display higher content of antimicrobial compounds. Numerous studies which subjected inoculated plants to varying degree of stresses report that the beneficial effect of PGP bacteria can increase as the growing conditions worsen, thanks to their capacity of decreasing the damage in plants [82,83,84]. As soil degradation and climate change are likely to aggravate the growing conditions of crops in many areas of the world, our study confirms that PGP bacteria can be a valuable resource to still achieve profitable and sustainable agriculture in the future.

## Data Availability

As all the relevant data are provided in the main text or the Supplementary Materials.

## Supplementary Materials

The following are available online at https://www.mdpi.com/article/10.3390/metabo11060358/s1, Figure S1: Principal component analysis of the polar metabolite profiles of Azospirillum-inoculated (B) and non-inoculated roots (C) of Brachypodium harvested at 7, 14, and 21 DAI. Ellipses around samples display 95% confidence areas, Figure S2: Volcano plots of significantly affected polar metabolites in the comparison between bacteria inoculated and non-inoculated samples at 7 DAI (a), 14 DAI (b) and 21 DAI (c). Red dots represent compounds with FC <−1.5 or >1.5 and probability of significant difference between mean of Azospirillum-inoculated and non-inoculated plants based on Student’s *t*-test *p* < 0.05, Figure S3: Principal component analysis of the lipid profiles of Azospirillum-inoculated (B) and non-inoculated roots (C) of Brachypodium harvested at 7, 14, and 21 DAI. Ellipses around samples display 95% confidence areas, Figure S4: Hierarchical clustering coupled with heatmap of the unidentified feature profiles of Azospirillum-inoculated (B) and non-inoculated roots (C) of Brachypodium harvested at 7, 14, and 21 DAI. The lettering at the bottom of the heatmap indicates the replicates: for each replicate, blue and red colors indicate the lower or higher abundance of specific lipids compared to the other replicates, with darker colors indicating more pronounced differences, Figure S5: Volcano plots of significantly affected lipids in the comparison between bacteria inoculated and non-inoculated samples at 7 DAI (a), 14 DAI (b), and 21 DAI (c). Red dots represent compounds with FC < −1.5 or >1.5 and probability of significant difference between mean of Azospirillum-inoculated and non-inoculated plants based on Student’s *t*-test *p* < 0.05, Figure S6: Relative response of Sucrose in roots of plants with or without Azospirillum inoculation harvested at 7, 14, and 21 DAI. Means are presented. *n* = 6 for all pooled samples except for non-inoculated at 21 DAI (*n* = 8), Figure S7: Relative response of γ-aminobutyric acid in roots of plants with or without Azospirillum inoculation harvested at 7, 14, and 21 DAI. Means are presented. *n* = 6 for all pooled samples except for non-inoculated at 21 DAI (*n* = 8), Figure S8. Relative response of Pi-Cer in roots of plants with or without Azospirillum inoculation harvested at 7, 14, and 21 DAI. Means are presented. *n* = 6 for all pooled samples except for non-inoculated at 21 DAI (*n* = 8), Table S1: List of polar metabolites detected in Brachypodium roots harvested at 7, 14, and 21 DAI. Columns display in ascending order the class of each compound, its name, the relative response fold changes (FC) (Azospirillum inoculated/non-inoculated plants) and the related *p*-value for each time point. Compounds are divided into amino acids (AA), organic acids (OA) sugars (SU) or “Others” if they did not belong to any of the previous classes. Compounds with a FC < −1.5 or FC > 1.5 and *p* < 0.05 are presented in red font, Table S2: (a) Features detected with LC-MS analysis of Azospirillum-inoculated (B) and non-inoculated (C) *Brachypodium* roots harvested at 7, 14, and 21 DAI. Average retention time (Rt) and mass to charge ratio (*m/z*) of each compound are presented. Columns D to AO report the relative level of detected features, with darker colors indicating higher abundance compared to the other replicates. ADGGA: acyl diacylglyceryl glucuronide, Cer-AP: ceramide alpha-hydroxy fatty acid-phytospingosine, DG: diacylglycerol, DGDG: digalactosyldiacylglycerol, DGGA: diacylglyceryl glucuronide, HexCer: hexosylceramide, HexCer-AP: hexosylceramide alpha-hydroxy fatty acid-phytospingosine, LPC: lysophophatidylcholine, MGDG: monogalactosyldiacylglycerol, PC: phophatidylcholine, PE: phosphatidylethanolamine, PG: phosphatidylglycerol, PI: phosphatidylinositol, Pi-Cer: ceramide phosphoinositol, TG: triacylglycerol. (b) List of glycerophospholipids and galactolipids detected in Brachypodium roots harvested at 7 and 21 DAI. Columns display the relative response fold changes (FC) (21 DAI/7 DAI) and the related *p*-value for each treatment. Red and blue font indicate a FC < −1.5 or >1.5 respectively, and probability of significant difference between time points based on Student’s *t*-test *p* < 0.05. DGDG: digalactosyldiacylglycerol, LPC: lysophosphatidylcholine, MGDG: monogalactosyldiacylglycerol, PC: phosphatidylcholine, PE: phosphatidylethanolamine, PG: phosphatidylglycerol, PI: phosphatidylinositol, Pi-Cer: ceramide phosphoinositol, (c) Composition and similarity of the K-means clusters of lipid species extracted from Azospirillum-inoculated and non-inoculated Brachypodium roots harvested at 7, 14, and 21 DAI. ADGGA: acyl diacylglyceryl glucuronide, Cer-AP: ceramide alpha-hydroxy fatty acid-phytospingosine, DG: diacylglycerol, DGDG: digalactosyldiacylglycerol, DGGA: diacylglyceryl glucuronide, HexCer: hexosylceramide, HexCer-AP: hexosylceramide alpha-hydroxy fatty acid-phytospingosine, LPC: lysophophatidylcholine, MGDG: monogalactosyldiacylglycerol, PC: phophatidylcholine, PE: phosphatidylethanolamine, PG: phosphatidylglycerol, PI: phosphatidylinositol, Pi-Cer: ceramide phosphoinositol, TG: triacylglycerol.

## Appendix A

**Table A1:** Reports on compounds affected in our study, in relation to plant response to stress and/or interaction with PGP bacteria.

Metabolite	Stress	Plant	PGP Bacteria Interaction	Role(s)
Aminocaproic acid	salinity	barley [85]	no	unknown
Campesterol	low temperature	rice [86,87], Maize [88]	no	brassinosteroids precursor
Campesterol	microbial attack	rice [27], barley [25]	no	brassinolide precursor
Diethylene glycol	Pb toxicity	*Sedum alfredii* [89]	no	unknown
Diethylene glycol	drought	*Nicotiana benthamiana* [90]	no	unknown
Glycerol-3-phosphate	pathogen attack	wheat [91]	no	systemic acquired resistance induction
Hexacosanol	pathogen attack	Asterids [92,93]	no	unknown
Hexacosanol	pathogen attack	*A. thaliana* [23]	no	wax barriers formation
Malic acid	P deficiency	wheat [40], barley [5], maize [94]	no	substrate P mobilization
Pantothenic acid	waterlogging	cucumber [95]	no	unknown
Pantothenic acid	drought	sorghum [96]	no	unknown
Pentonic acid, 1,4-lactone	low temperature	*A. thaliana* [97]	no	unknown
Pentonic acid, 1,4-lactone	P deficiency	*Camelia sinensis* [49]	no	unknown
Putrescine	P deficiency	rice [98]	no	growth inhibition
Pyroglutamic acid	no	*Lolium multiflorum* [99], tomato [100]	*Pseudomonas putida* [99], *Pseudomonas fluorescens* [100]	C source for PGP bacteria [99], bacterial chemotaxis [100]
Pyroglutamic acid	drought	lettuce [101]	no	photosynthetic rate improvement, ROS scavenging
Ribonic acid	no	sugarcane [102]	*Herbaspirillum seropedicae, Gluconacetobacter diazotrophicus*	unknown
Ribonic acid	P deficiency	*Camelia sinensis* [49], oat [103]	no	unknown
Serotonin	nutrient deficiency	rice [50]	no	ROS scavenging, nutrient recycling
Serotonin	no	barley [104]	no	auxin metabolism modification
Sinapic acid	pathogen attack	*Aegilops variabilis* [44]	no	unknown
Sinapic acid	no	wheat [43]	no	Antioxidant and antimicrobial activity
Trehalose	no	Maize [14]	*Herbaspirillum seropedicae, Azospirillum brasilense*	signaling during the interaction with PGP bacteria
Trehalose	drought	maize [105]	genetically modified *A. brasilense*	osmotic stress tolerance, root elongation
Trehalose	P deficiency	rice [106]	no	root elongation
Xylose	no	rice [107]	*Corynebacterium* sp., *Rhizobium* sp.	C source for PGP bacteria
Xylose	salinity	Wheat [108]	no	unknown
α-ketoglutaric acid	P deficiency	rice [109]	no	unknown

## References

[1] Schillaci M., Arsova B., Walker R., Smith P.M.C., Nagel K.A., Roessner U., Watt M. (2021). Time-resolution of the shoot and root growth of the model cereal Brachypodium in response to inoculation with Azospirillum bacteria at low phosphorus and temperature. Plant Growth Regul..

[2] Chinnusamy V., Zhu J., Zhu J.-K. (2007). Cold stress regulation of gene expression in plants. Trends Plant Sci..

[3] Strand Å., Hurry V., Henkes S., Huner N., Gustafsson P., Gardeström P., Stitt M. (1999). Acclimation of Arabidopsis Leaves Developing at Low Temperatures. Increasing Cytoplasmic Volume Accompanies Increased Activities of Enzymes in the Calvin Cycle and in the Sucrose-Biosynthesis Pathway. Plant Physiol..

[4] Plaxton W.C., Tran H.T. (2011). Metabolic Adaptations of Phosphate-Starved Plants. Plant Physiol..

[5] Huang C.Y., Roessner U., Eickmeier I., Genc Y., Callahan D.L., Shirley N., Langridge P., Bacic A. (2008). Metabolite Profiling Reveals Distinct Changes in Carbon and Nitrogen Metabolism in Phosphate-Deficient Barley Plants (*Hordeum vulgare* L.). Plant Cell Physiol..

[6] Grant C.A., Flaten D.N., Tomasiewicz D.J., Sheppard S.C. (2001). The importance of early season phosphorus nutrition. Can. J. Plant Sci..

[7] Sharkey T.D., Stitt M., Heineke D., Gerhardt R., Raschke K., Heldt H.W. (1986). Limitation of photosynthesis by carbon metabolism: II. O_2_-insensitive CO_2_ uptake results from limitation of triose phosphate utilization. Plant Physiol..

[8] Hurry V., Strand Å., Furbank R., Stitt M. (2000). The role of inorganic phosphate in the development of freezing tolerance and the acclimatization of photosynthesis to low temperature is revealed by the pho mutants of *Arabidopsis thaliana*. Plant J..

[9] Schlüter U., Colmsee C., Scholz U., Bräutigam A., Weber A.P.M., Zellerhoff N., Bucher M., Fahnenstich H., Sonnewald U. (2013). Adaptation of maize source leaf metabolism to stress related disturbances in carbon, nitrogen and phosphorus balance. BMC Genom..

[10] Aeron A., Kumar S., Pandey P., Maheshwari D. (2011). Emerging role of plant growth promoting rhizobacteria in agrobiology. Bacteria in Agrobiology: Crop Ecosystems.

[11] Glick B.R. Promotion of plant growth by soil bacteria that regulate plant ethylene levels. Proceedings of the 33rd Annual Meeting of the Plant Growth Regulation Society of America.

[12] Goswami D., Thakker J., Dhandhukia P.C. (2016). Portraying mechanics of plant growth promoting rhizobacteria (PGPR): A review. Cogent Food Agric..

[13] Agtuca B.J., Stopka S.A., Tuleski T.R., Amaral F.P.D., Evans S., Liu Y., Xu D., Monteiro R.A., Koppenaal D.W., Paša-Tolić L. (2020). In-Situ Metabolomic Analysis of *Setaria viridis* Roots Colonized by Beneficial Endophytic Bacteria. Mol. Plant Microbe Interact..

[14] Brusamarello-Santos L.C., Gilard F., Brulé L., Quilleré I., Gourion B., Ratet P., de Souza E.M., Lea P.J., Hirel B. (2017). Metabolic profiling of two maize (*Zea mays* L.) inbred lines inoculated with the nitrogen fixing plant-interacting bacteria *Herbaspirillum seropedicae* and *Azospirillum brasilense*. PLoS ONE.

[15] Chamam A., Sanguin H., Bellvert F., Meiffren G., Comte G., Wisniewski-Dyé F., Bertrand C., Prigent-Combaret C. (2013). Plant secondary metabolite profiling evidences strain-dependent effect in the *Azospirillum*–*Oryza sativa* association. Phytochemistry.

[16] Curzi M.J., Ribaudo C.M., Trinchero G.D., Curá J.A., Pagano E.A. (2008). Changes in the content of organic and amino acids and ethylene production of rice plants in response to the inoculation with *Herbaspirillum seropedicae*. J. Plant Interact..

[17] Valette M., Rey M., Gerin F., Comte G., Wisniewski-Dyé F. (2020). A common metabolomic signature is observed upon inoculation of rice roots with various rhizobacteria. J. Integr. Plant Biol..

[18] Gagné-Bourque F., Bertrand A., Claessens A., Aliferis K.A., Jabaji S. (2016). Alleviation of Drought Stress and Metabolic Changes in Timothy (*Phleum pratense* L.) Colonized with Bacillus subtilis B26. Front. Plant Sci..

[19] Planchamp C., Glauser G., Mauch-Mani B. (2015). Root inoculation with *Pseudomonas putida* KT2440 induces transcriptional and metabolic changes and systemic resistance in maize plants. Front. Plant Sci..

[20] Catalan P., Chalhoub B., Chochois V., Garvin D.F., Hasterok R., Manzaneda A.J., Mur L.A., Pecchioni N., Rasmussen S.K., Vogel J.P. (2014). Update on the genomics and basic biology of *Brachypodium*: International *Brachypodium* Initiative (IBI). Trends Plant Sci..

[21] Rothballer M., Schmid M., Fekete A., Hartmann A. (2005). Comparative in situ analysis of ipdC-gfpmut3 promoter fusions of *Azospirillum brasilense* strains Sp7 and Sp245. Environ. Microbiol..

[22] Plaxton W., Carswell M.C. (1999). Metabolic aspects of the phosphate starvation response in plants. Plant Responses to Environmental Stresses.

[23] Shanmugarajah K., Linka N., Gräfe K., Smits S.H.J., Weber A.P.M., Zeier J., Schmitt L. (2019). ABCG1 contributes to suberin formation in *Arabidopsis thaliana* roots. Sci. Rep..

[24] Zimmermann W., Eeemüller E. (1984). Degradation of Raspberry Suberin by *Fusarium solani* f. sp. *Pisi* and *Armillaria mellea*. J. Phytopathol..

[25] Ali S.S., Kumar G.B.S., Khan M., Doohan F. (2013). Brassinosteroid Enhances Resistance to Fusarium Diseases of Barley. Phytopathology.

[26] Bajguz A., Hayat S. (2009). Effects of brassinosteroids on the plant responses to environmental stresses. Plant Physiol. Biochem..

[27] Nakashita H., Yasuda M., Nitta T., Asami T., Fujioka S., Arai Y., Sekimata K., Takatsuto S., Yamaguchi I., Yoshida S. (2003). Brassinosteroid functions in a broad range of disease resistance in tobacco and rice. Plant J..

[28] Chanda B., Xia Y., Mandal M.K., Yu K., Sekine K.-T., Gao Q.-M., Selote D., Hu Y., Stromberg A., Navarre D. (2011). Glycerol-3-phosphate is a critical mobile inducer of systemic immunity in plants. Nat. Genet..

[29] Shah J., Zeier J. (2013). Long-distance communication and signal amplification in systemic acquired resistance. Front. Plant Sci..

[30] Yokota T. (1997). The structure, biosynthesis and function of brassinosteroids. Trends Plant Sci..

[31] Yang C.-J., Zhang C., Lu Y.-N., Jin J.-Q., Wang X.-L. (2011). The Mechanisms of Brassinosteroids’ Action: From Signal Transduction to Plant Development. Mol. Plant.

[32] Zhu J., Lynch J.P. (2004). The contribution of lateral rooting to phosphorus acquisition efficiency in maize (*Zea mays*) seedlings. Funct. Plant Biol..

[33] Lunn J.E., Delorge I., Figueroa C.M., Van Dijck P., Stitt M. (2014). Trehalose metabolism in plants. Plant J..

[34] Griffiths C.A., Paul M.J., Foyer C.H. (2016). Metabolite transport and associated sugar signalling systems underpinning source/sink interactions. Biochim. Biophys. Acta Bioenerg..

[35] Singh A., Kumar P., Gautam V., Rengasamy B., Adhikari B., Udayakumar M., Sarkar A.K. (2016). Root transcriptome of two contrasting indica rice cultivars uncovers regulators of root development and physiological responses. Sci. Rep..

[36] Hammond J.P., White P.J. (2011). Sugar Signaling in Root Responses to Low Phosphorus Availability. Plant Physiol..

[37] Rasmussen S., Parsons A.J., Jones C.S. (2012). Metabolomics of forage plants: A review. Ann. Bot..

[38] Harrold S., Tabatabai M. (2006). Release of inorganic phosphorus from soils by low-molecular-weight organic acids. Commun. Soil Sci. Plant Anal..

[39] Hocking P.J. (2001). Organic acids exuded from roots in phosphorus uptake and aluminum tolerance of plants in acid soils. Adv. Agron..

[40] Neumann G., Römheld V. (1999). Root excretion of carboxylic acids and protons in phosphorus-deficient plants. Plant Soil.

[41] Jacoby R.P., Millar A.H., Taylor N.L. (2013). Investigating the Role of Respiration in Plant Salinity Tolerance by Analyzing Mitochondrial Proteomes from Wheat and a Salinity-Tolerant Amphiploid (Wheat × *Lophopyrum elongatum*). J. Proteome Res..

[42] Morgan P.W., Drew M.C. (1997). Ethylene and plant responses to stress. Physiol. Plant..

[43] Jeong E.-Y., Sung B.-K., Song H.-Y., Yang J.-Y., Kim D.-K., Lee H.-S. (2010). Antioxidative and Antimicrobial Activities of Active Materials Derived from *Triticum aestivum* Sprouts. J. Korean Soc. Appl. Biol. Chem..

[44] Huang Q., Li L., Zheng M., Chen F., Long H., Deng G., Pan Z., Liang J., Li Q., Yu M. (2018). The Tryptophan decarboxylase 1 Gene from *Aegilops variabilis* No.1 Regulate the Resistance against Cereal Cyst Nematode by Altering the Downstream Secondary Metabolite Contents rather than Auxin Synthesis. Front. Plant Sci..

[45] Selvi K., Paul J., Vijaya V., Saraswathi K. (2017). Analyzing the efficacy of phosphate solubilizing microorganisms by enrichment culture techniques. Biochem. Mol. Biol. J..

[46] Echevarría C., Maurĩno S.G., Maldonado J.M. (1984). Reversible inactivation of maize leaf nitrate reductase. Phytochemistry.

[47] Machingura M., Salomon E., Jez J.M., Ebbs S.D. (2016). The β-cyanoalanine synthase pathway: Beyond cyanide detoxification. Plant Cell Environ..

[48] Dias D., Hill C.B., Jayasinghe N.S., Atieno J., Sutton T., Roessner U. (2015). Quantitative profiling of polar primary metabolites of two chickpea cultivars with contrasting responses to salinity. J. Chromatogr. B.

[49] Ding Z., Jia S., Wang Y., Xiao J., Zhang Y. (2017). Phosphate stresses affect ionome and metabolome in tea plants. Plant Physiol. Biochem..

[50] Kang K., Kim Y.-S., Park S., Back K. (2009). Senescence-Induced Serotonin Biosynthesis and Its Role in Delaying Senescence in Rice Leaves. Plant Physiol..

[51] Li Z., Yu J., Peng Y., Huang B. (2017). Metabolic pathways regulated by abscisic acid, salicylic acid and γ-aminobutyric acid in association with improved drought tolerance in creeping bentgrass (*Agrostis stolonifera*). Physiol. Plant..

[52] Pacovsky R.S. (1988). Influence of inoculation with *Azospirillum brasilense* and *Glomus fasciculatum* on sorghum nutrition. Plant Soil.

[53] Ramakrishna A., Giridhar P., Ravishankar G.A. (2011). Phytoserotonin: A review. Plant Signal. Behav..

[54] Hayashi K., Fujita Y., Ashizawa T., Suzuki F., Nagamura Y., Hayano-Saito Y. (2016). Serotonin attenuates biotic stress and leads to lesion browning caused by a hypersensitive response to *Magnaporthe oryzae* penetration in rice. Plant J..

[55] Pelagio-Flores R., Ortíz-Castro R., Méndez-Bravo A., Macías-Rodríguez L., López-Bucio J. (2011). Serotonin, a Tryptophan-Derived Signal Conserved in Plants and Animals, Regulates Root System Architecture Probably Acting as a Natural Auxin Inhibitor in *Arabidopsis thaliana*. Plant Cell Physiol..

[56] Wan J., Zhang P., Sun L., Li S., Wang R., Zhou H., Wang W., Xu J. (2018). Involvement of reactive oxygen species and auxin in serotonin-induced inhibition of primary root elongation. J. Plant Physiol..

[57] Tjellström H., Andersson M.X., Larsson K.E., Sandelius A.S. (2008). Membrane phospholipids as a phosphate reserve: The dynamic nature of phospholipid-to-digalactosyl diacylglycerol exchange in higher plants. Plant Cell Environ..

[58] Yu B., Xu C., Benning C. (2002). Arabidopsis disrupted in *SQD2* encoding sulfolipid synthase is impaired in phosphate-limited growth. Proc. Natl. Acad. Sci. USA.

[59] Kelly A.A., Dörmann P. (2002). *DGD2*, an *Arabidopsis* Gene Encoding a UDP-Galactose-dependent Digalactosyldiacylglycerol Synthase Is Expressed during Growth under Phosphate-limiting Conditions. J. Biol. Chem..

[60] Zhang G., Ahmad M.Z., Chen B., Manan S., Zhang Y., Jin H., Wang X., Zhao J. (2020). Lipidomic and transcriptomic profiling of developing nodules reveals the essential roles of active glycolysis and fatty acid and membrane lipid biosynthesis in soybean nodulation. Plant J..

[61] Abeer H., Abdallah E., Alqarawi A., Al-Huqail A.A., Alshalawi S., Wirth S., Dilfuza E. (2015). Impact of plant growth promoting *Bacillus subtilis* on growth and physiological parameters of *Bassia indica* (Indian bassia) grown udder salt stress. Pak. J. Bot..

[62] Calderon-Vazquez C., Ibarra-Laclette E., Caballero-Perez J., Herrera-Estrella L.R. (2008). Transcript profiling of *Zea mays* roots reveals gene responses to phosphate deficiency at the plant- and species-specific levels. J. Exp. Bot..

[63] Siebers M., Brands M., Wewer V., Duan Y., Hölzl G., Dörmann P. (2016). Lipids in plant-microbe interactions. Biochim. Biophys. Acta Mol. Cell Biol. Lipids.

[64] Cao D., Lutz A., Hill C.B., Callahan D.L., Roessner U. (2017). A Quantitative Profiling Method of Phytohormones and Other Metabolites Applied to Barley Roots Subjected to Salinity Stress. Front. Plant Sci..

[65] Pereyra M., Zalazar C., Barassi C. (2006). Root phospholipids in *Azospirillum*-inoculated wheat seedlings exposed to water stress. Plant Physiol. Biochem..

[66] Gika H.G., Theodoridis G.A., Wilson I.D., Theodoridis G., Gika H., Wilson I. (2018). Metabolic Profiling: Status, Challenges, and Perspective. Metabolic Profiling. Methods in Molecular Biology.

[67] Dickinson E., Rusilowicz M.J., Dickinson M., Charlton A.J., Bechtold U., Mullineaux P.M., Wilson J. (2018). Integrating transcriptomic techniques and *k*-means clustering in metabolomics to identify markers of abiotic and biotic stress in *Medicago truncatula*. Metabolomics.

[68] Gioia T., Galinski A., Lenz H., Müller C., Lentz J., Heinz K., Briese C., Putz A., Fiorani F., Watt M. (2017). GrowScreen-PaGe, a non-invasive, high-throughput phenotyping system based on germination paper to quantify crop phenotypic diversity and plasticity of root traits under varying nutrient supply. Funct. Plant Biol..

[69] Hill C.B., Roessner U. (2013). Metabolic profiling of plants by GC-MS. The Handbook of Plant Metabolomics: Metabolite Profiling and Networking.

[70] Cheong B.E., Onyemaobi O., Ho W.W.H., Ben Biddulph T., Rupasinghe T.W.T., Roessner U., Dolferus R. (2020). Phenotyping the Chilling and Freezing Responses of Young Microspore Stage Wheat Spikes Using Targeted Metabolome and Lipidome Profiling. Cells.

[71] Shiva S., Enninful R., Roth M.R., Tamura P., Jagadish K., Welti R. (2018). An efficient modified method for plant leaf lipid extraction results in improved recovery of phosphatidic acid. Plant Methods.

[72] Kehelpannala C., Rupasinghe T.W.T., Hennessy T., Bradley D., Ebert B., Roessner U. (2020). A comprehensive comparison of four methods for extracting lipids from Arabidopsis tissues. Plant Methods.

[73] Hillyer K.E., Dias D., Lutz A., Wilkinson S.P., Roessner U., Davy S.K. (2017). Metabolite profiling of symbiont and host during thermal stress and bleaching in the coral *Acropora aspera*. Coral Reefs.

[74] Kehelpannala C., Rupasinghe T., Pasha A., Esteban E., Hennessy T., Bradley D., Ebert B., Provart N.J., Roessner U. (2021). An Arabidopsis lipid map reveals differences between tissues and dynamic changes throughout development. Plant J..

[75] Tsugawa H., Cajka T., Kind T., Ma Y., Higgins B.T., Ikeda K., Kanazawa M., VanderGheynst J.S., Fiehn O., Arita M. (2015). MS-DIAL: Data-independent MS/MS deconvolution for comprehensive metabolome analysis. Nat. Methods.

[76] Yu D., Rupasinghe T.W., Boughton B.A., Natera S.H., Hill C.B., Tarazona P., Feussner I., Roessner U. (2018). A high-resolution HPLC-QqTOF platform using parallel reaction monitoring for in-depth lipid discovery and rapid profiling. Anal. Chim. Acta.

[77] Ihaka R., Gentleman R. (1996). R: A language for data analysis and graphics. J. Comput. Graph. Stat..

[78] Gu Z., Eils R., Schlesner M. (2016). Complex heatmaps reveal patterns and correlations in multidimensional genomic data. Bioinformatics.

[79] Valero-Mora P.M. (2010). ggplot2: Elegant graphics for data analysis. J. Stat. Softw..

[80] Kawasaki A., Donn S., Ryan P.R., Mathesius U., Devilla R., Jones A., Watt M. (2016). Microbiome and Exudates of the Root and Rhizosphere of *Brachypodium distachyon*, a Model for Wheat. PLoS ONE.

[81] Olanrewaju O.S., Ayangbenro A.S., Glick B.R., Babalola O.O., Ayangbenro A. (2019). Plant health: Feedback effect of root exudates-rhizobiome interactions. Appl. Microbiol. Biotechnol..

[82] Casanovas E.M., Barassi C.A., Sueldo R.J. (2002). Azospirillum inoculation mitigates water stress effects in maize seedlings. Cereal Res. Commun..

[83] Hardoim P.R., Van Overbeek L.S., Berg G., Pirttilä A.M., Compant S., Campisano A., Döring M., Sessitsch A. (2015). The hidden world within plants: Ecological and evolutionary considerations for defining functioning of microbial endophytes. Microbiol. Mol. Biol. Rev..

[84] Yuwono T., Handayani D., Soedarsono J. (2005). The role of osmotolerant rhizobacteria in rice growth under different drought conditions. Aust. J. Agric. Res..

[85] Wang Y., Zeng X., Xu Q., Mei X., Yuan H., Jiabu D., Sang Z., Nyima T. (2019). Metabolite profiling in two contrasting Tibetan hulless barley cultivars revealed the core salt-responsive metabolome and key salt-tolerance biomarkers. AoB Plants.

[86] Fujii S., Saka H. (2001). The Promotive Effect of Brassinolide on Lamina Joint-Cell Elongation, Germination and Seedling Growth under Low-Temperature Stress in Rice (*Oryza sativa* L.). Plant Prod. Sci..

[87] Hotta Y., Tanaka T., Luo B., Takeuchi Y., Konnai M. (1998). Improvement of Cold Resistance in Rice Seedlings by 5-Aminolevulinic Acid. J. Pestic. Sci..

[88] He R.-Y., Wang G.-J., Wang X.-S. (1991). Effects of brassinolide on growth and chilling resistance of maize seedlings. Brassinosteroids.

[89] Luo Q., Wang S., Sun L.-N., Wang H., Bao T., Adeel M. (2017). Identification of root exudates from the Pb-accumulator *Sedum alfredii* under Pb stresses and assessment of their roles. J. Plant Interact..

[90] Dastogeer K.M.G., Li H., Sivasithamparam K., Jones M., Du X., Ren Y., Wylie S.J. (2017). Metabolic responses of endophytic *Nicotiana benthamiana* plants experiencing water stress. Environ. Exp. Bot..

[91] Yang Y., Zhao J., Liu P., Xing H., Li C., Wei G., Kang Z. (2013). Glycerol-3-Phosphate Metabolism in Wheat Contributes to Systemic Acquired Resistance against *Puccinia striiformis* f. sp. tritici. PLoS ONE.

[92] Castilho P., Savluchinske-Feio S., Weinhold T.S., Gouveia S.C. (2012). Evaluation of the antimicrobial and antioxidant activities of essential oils, extracts and their main components from oregano from Madeira Island, Portugal. Food Control.

[93] Mbosso E.J.T., Ngouela S., Nguedia J.C.A., Beng V.P., Rohmer M., Tsamo E. (2010). In vitro antimicrobial activity of extracts and compounds of some selected medicinal plants from Cameroon. J. Ethnopharmacol..

[94] Gaume A., Machler F., De Leon C., Narro L., Frossard E. (2001). Low-P tolerance by maize (*Zea mays* L.) genotypes: Significance of root growth, and organic acids and acid phosphatase root exudation. Plant Soil.

[95] Qi X.-H., Xu X.-W., Lin X.-J., Zhang W.-J., Chen X.-H. (2012). Identification of differentially expressed genes in cucumber (*Cucumis sativus* L.) root under waterlogging stress by digital gene expression profile. Genomics.

[96] Pavli O.I., Vlachos C.E., Kalloniati C., Flemetakis E., Skaracis G.N. (2013). Metabolite profiling reveals the effect of drought on sorghum (‘*Sorghum bicolor*’ L. Moench) metabolism. Plant Omics.

[97] Chen M., Thelen J.J. (2016). Acyl-lipid desaturase 1 primes cold acclimation response in *Arabidopsis*. Physiol. Plant..

[98] Shih C.Y., Kao C.H. (1996). Growth Inhibition in Suspension-Cultured Rice Cells under Phosphate Deprivation Is Mediated through Putrescine Accumulation. Plant Physiol..

[99] Kuiper I., Kravchenko L.V., Bloemberg G.V., Lugtenberg B.J.J. (2002). Pseudomonas putida Strain PCL1444, Selected for Efficient Root Colonization and Naphthalene Degradation, Effectively Utilizes Root Exudate Components. Mol. Plant Microbe Interact..

[100] De Weert S., Vermeiren H., Mulders I.H.M., Kuiper I., Hendrickx N., Bloemberg G.V., Vanderleyden J., De Mot R., Lugtenberg B.J.J. (2002). Flagella-Driven Chemotaxis towards Exudate Components Is an Important Trait for Tomato Root Colonization by *Pseudomonas fluorescens*. Mol. Plant Microbe Interact..

[101] Jiménez-Arias D., García-Machado F.J., Morales-Sierra S., Luis J.C., Suarez E., Hernández M., Valdés F., Borges A.A. (2019). Lettuce plants treated with L-pyroglutamic acid increase yield under water deficit stress. Environ. Exp. Bot..

[102] Aguiar N.O., Olivares F.L., Novotny E.H., Canellas L.P. (2018). Changes in metabolic profiling of sugarcane leaves induced by endophytic diazotrophic bacteria and humic acids. PeerJ.

[103] Wang Y., Lysøe E., Armarego-Marriott T., Erban A., Paruch L., Van Eerde A., Bock R., Liu-Clarke J. (2018). Transcriptome and metabolome analyses provide insights into root and root-released organic anion responses to phosphorus deficiency in oat. J. Exp. Bot..

[104] Csaba G., Pál K. (1982). Effects of insulin, triiodothyronine, and serotonin on plant seed development. Protoplasma.

[105] Rodríguez-Salazar J., Suárez R., Caballero-Mellado J., Iturriaga G. (2009). Trehalose accumulation in *Azospirillum brasilense* improves drought tolerance and biomass in maize plants. FEMS Microbiol. Lett..

[106] Tyagi W., Rai M. (2017). Root transcriptomes of two acidic soil adapted Indica rice genotypes suggest diverse and complex mechanism of low phosphorus tolerance. Protoplasma.

[107] Naher U., Radziah O., Halimi M., Shamsuddin Z., Razi I.M. (2008). Effect of Inoculation on Root Exudates Carbon Sugar and Amino Acids Production of Different Rice Varieties. Res. J. Microbiol..

[108] Guo R., Yang Z., Li F., Yan C., Zhong X., Liu Q., Xia X., Li H., Zhao L. (2015). Comparative metabolic responses and adaptive strategies of wheat (*Triticum aestivum*) to salt and alkali stress. BMC Plant Biol..

[109] Tawaraya K., Horie R., Saito A., Shinano T., Wagatsuma T., Saito K., Oikawa A. (2013). Metabolite profiling of shoot extracts, root extracts, and root exudates of rice plant under phosphorus deficiency. J. Plant Nutr..

