# Supplementary figures and images for: The Metabolic Response of Brachypodium Roots to the Interaction with Beneficial Bacteria Is Affected by the Plant Nutritional Status
Source: Metabolites. 2021 Jun 3;11(6):358. doi: 10.3390/metabo11060358 (PMC8228974; doi:10.3390/metabo11060358)

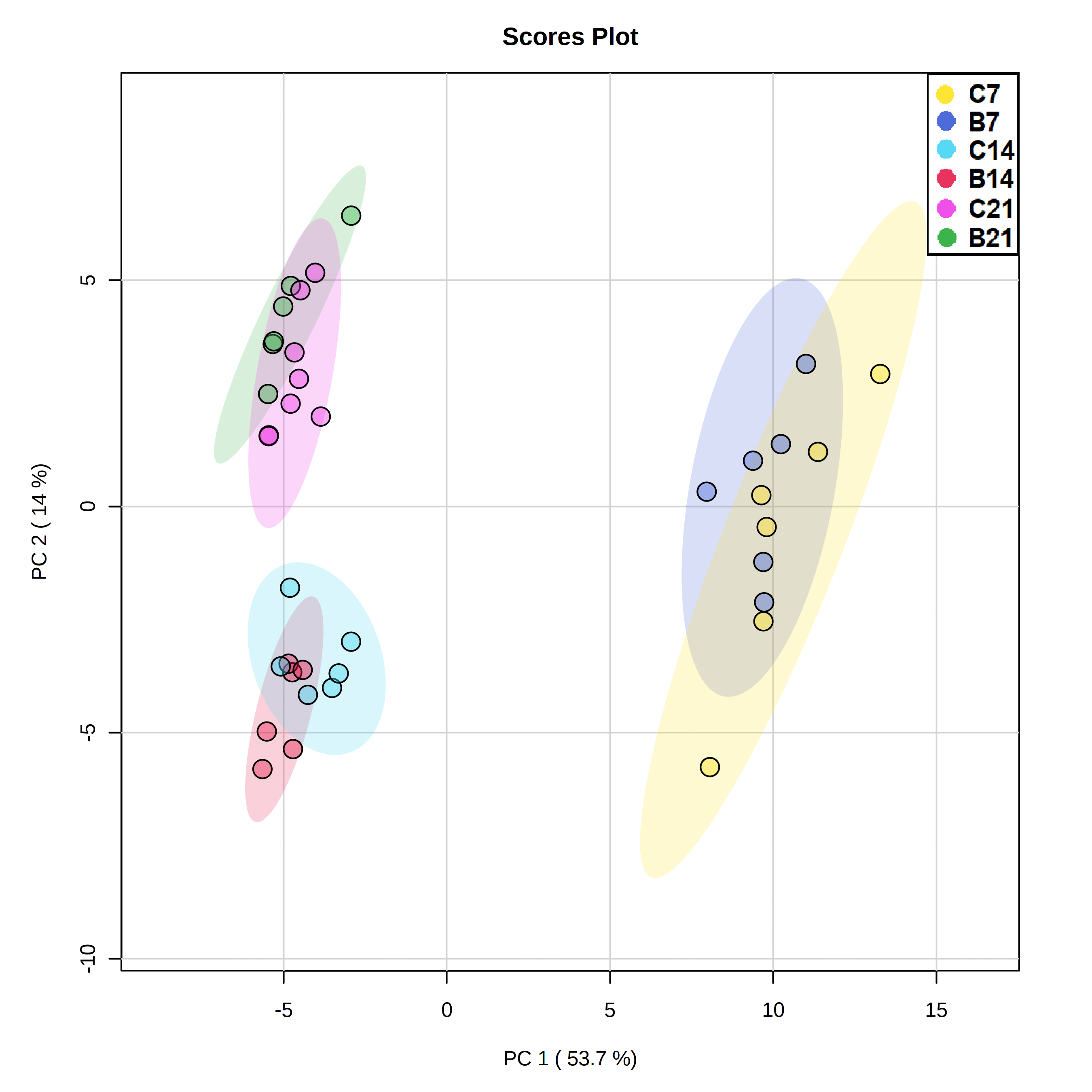

Supplement: Supplementary file 1 [file metabolites-11-00358-s001.zip › Figure S1.tif]

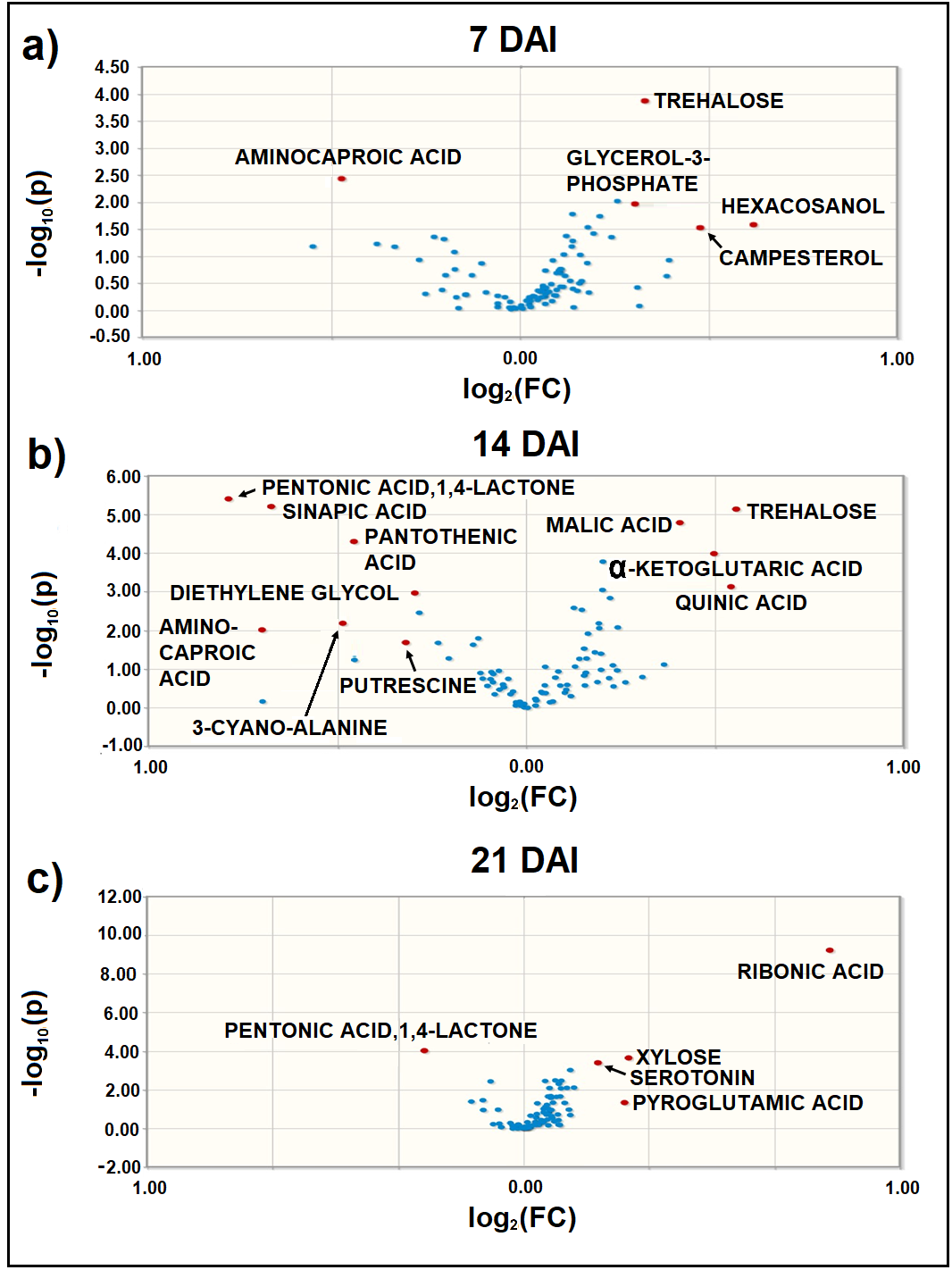

Supplement: Supplementary file 1 [file metabolites-11-00358-s001.zip › Figure S2.tif]

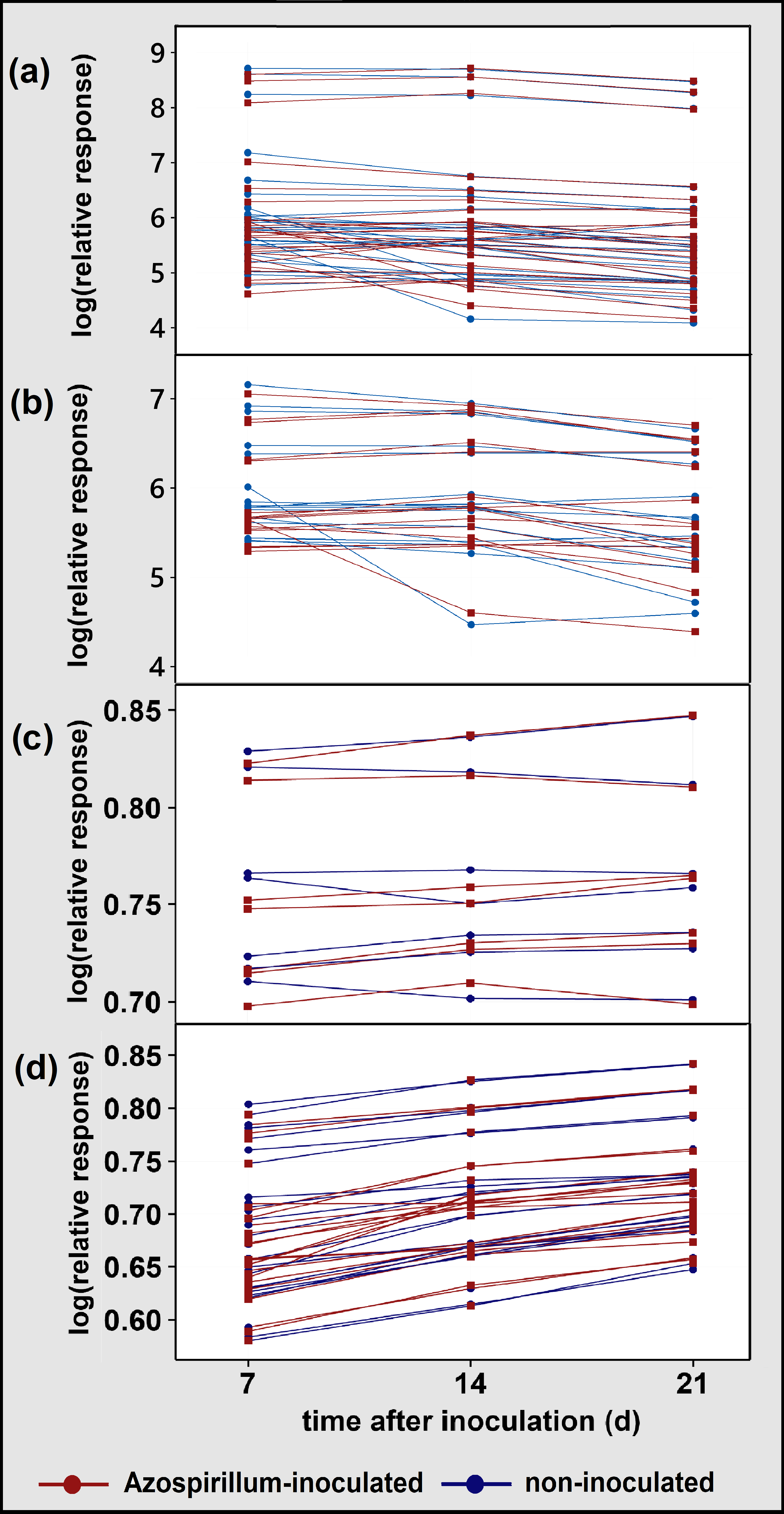

Supplement: Supplementary file 1 [file metabolites-11-00358-s001.zip › Figure S3.tif]

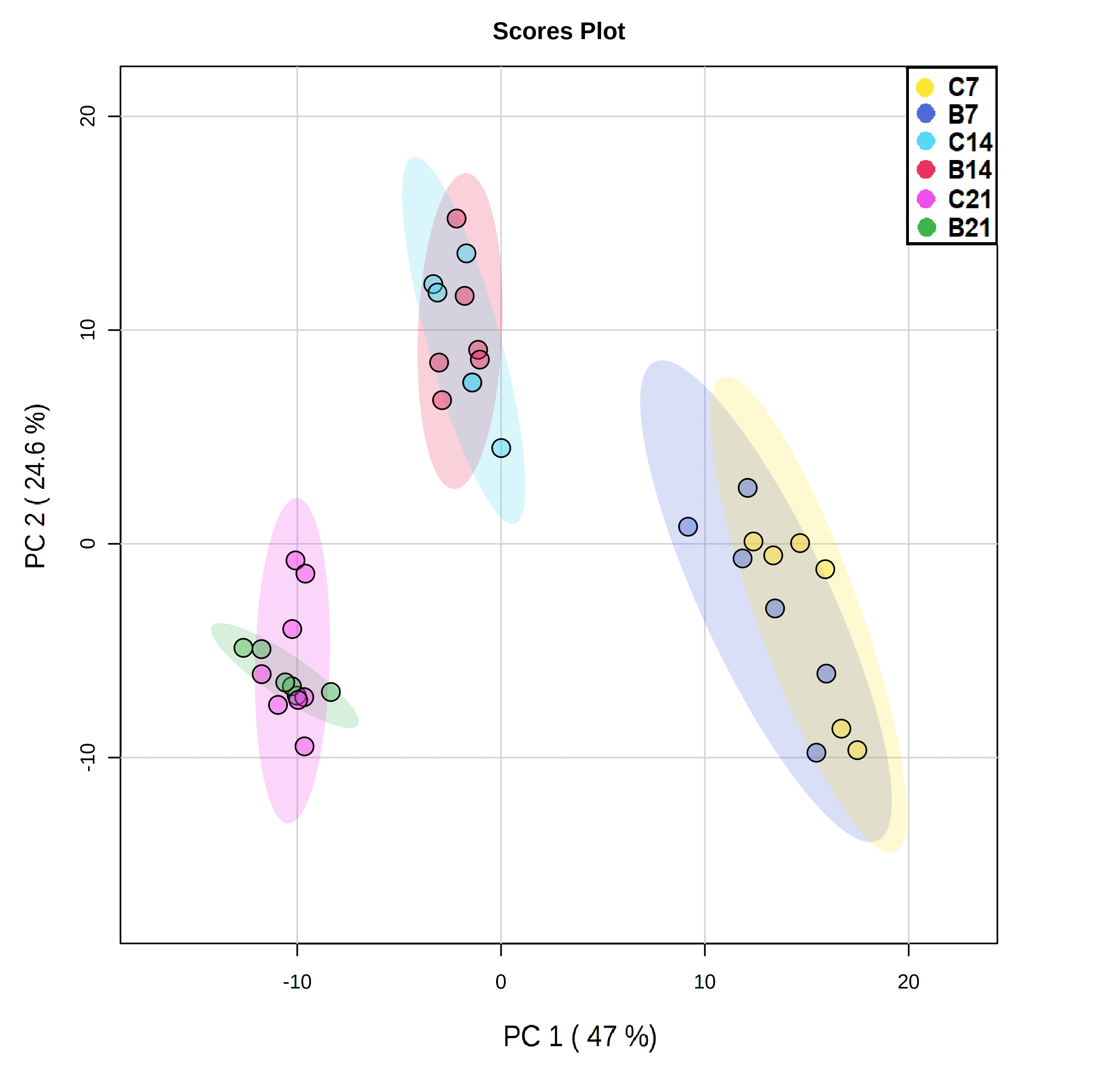

Supplement: Supplementary file 1 [file metabolites-11-00358-s001.zip › Figure S4.tif]

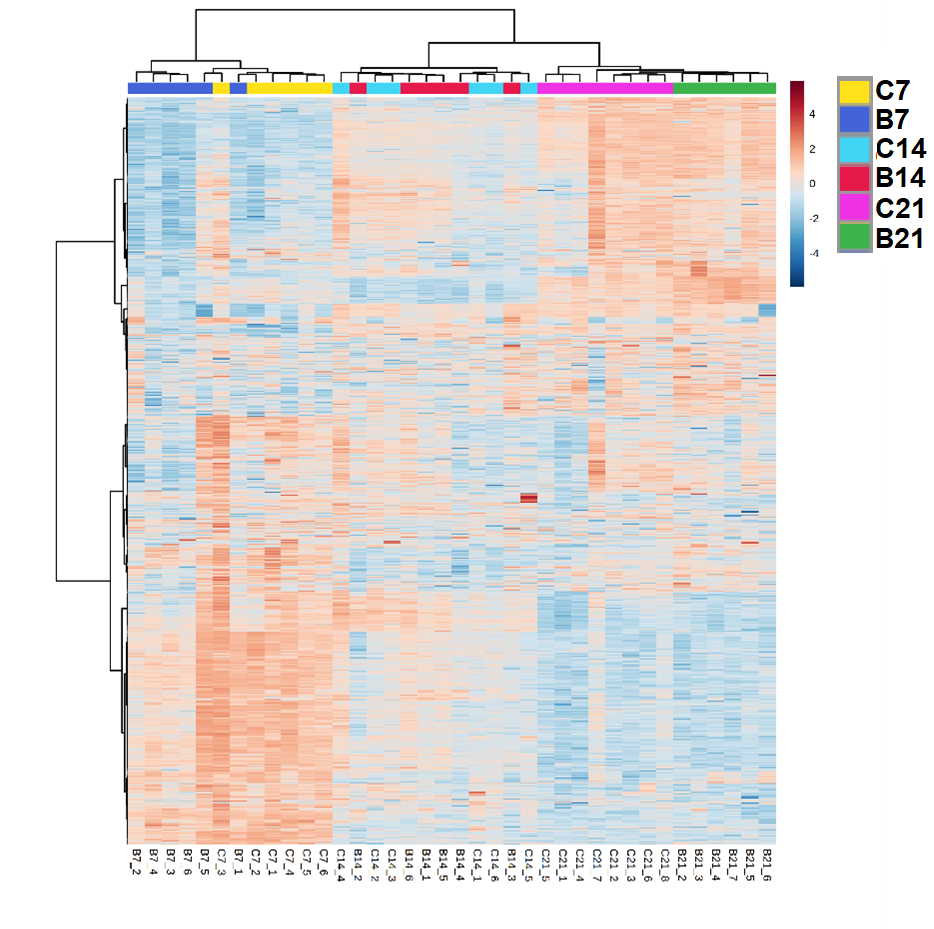

Supplement: Supplementary file 1 [file metabolites-11-00358-s001.zip › Figure S5.tif]

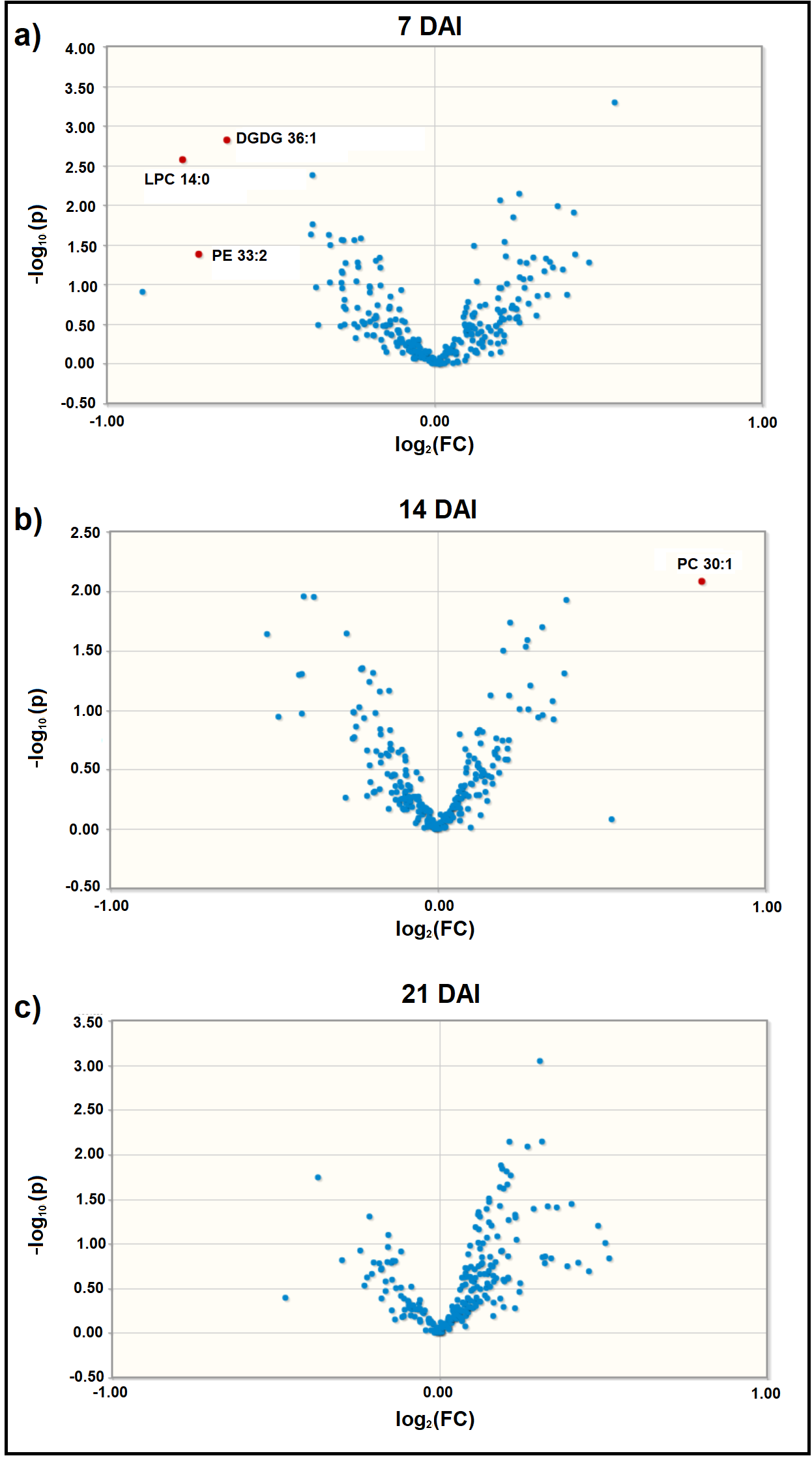

Supplement: Supplementary file 1 [file metabolites-11-00358-s001.zip › Figure S6.tif]

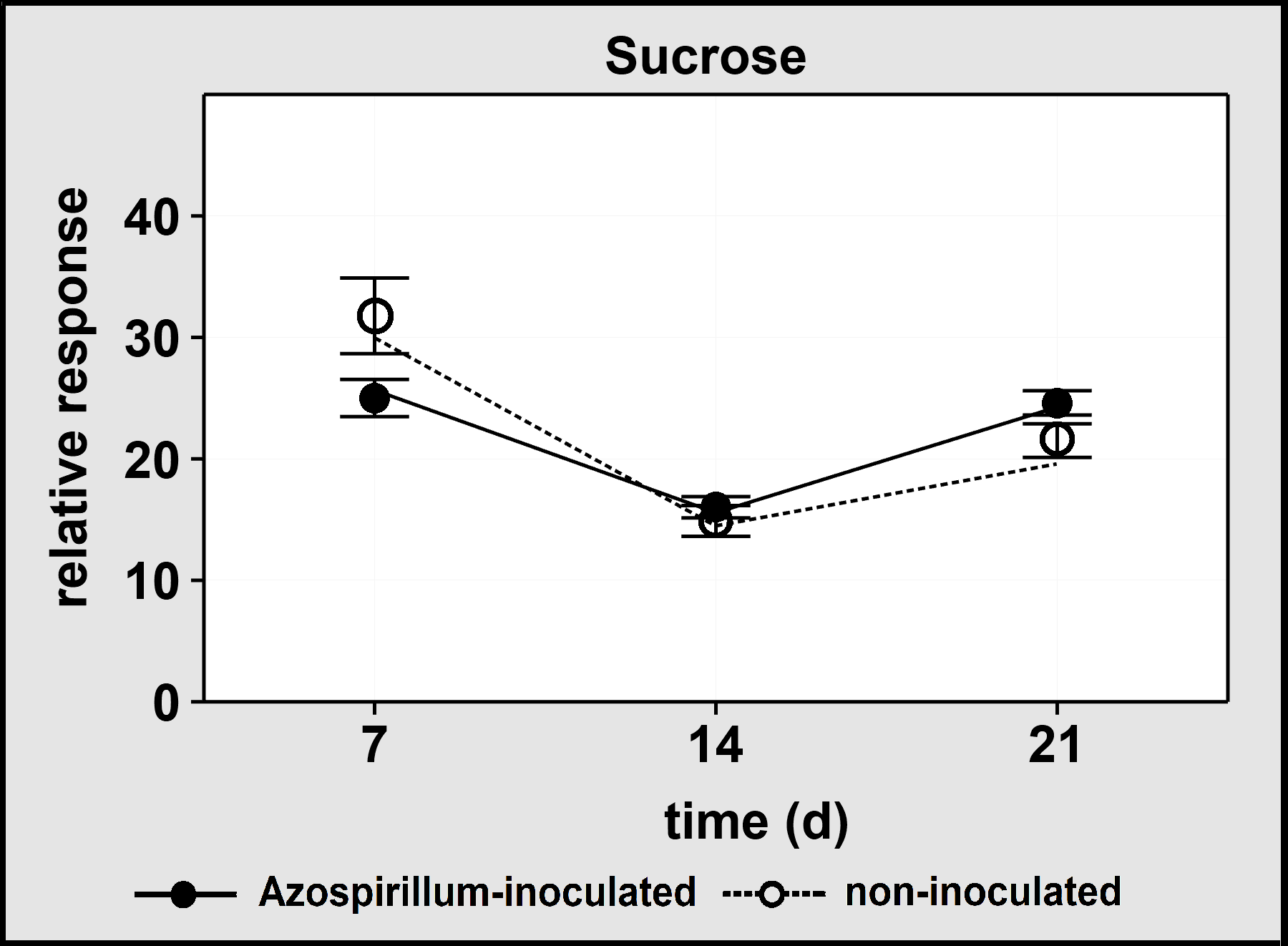

Supplement: Supplementary file 1 [file metabolites-11-00358-s001.zip › Figure S7.tif]

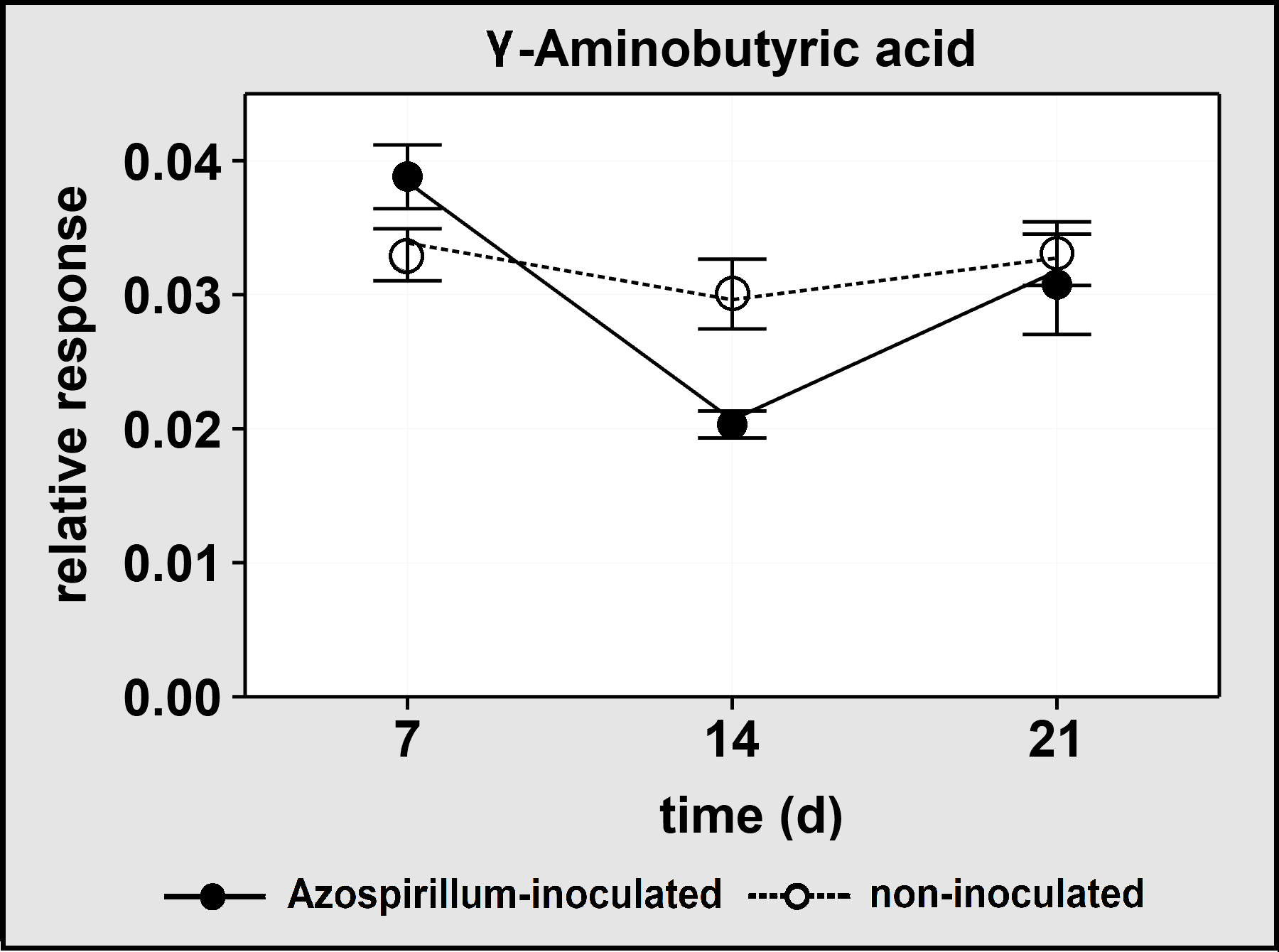

Supplement: Supplementary file 1 [file metabolites-11-00358-s001.zip › Figure S8.tif]

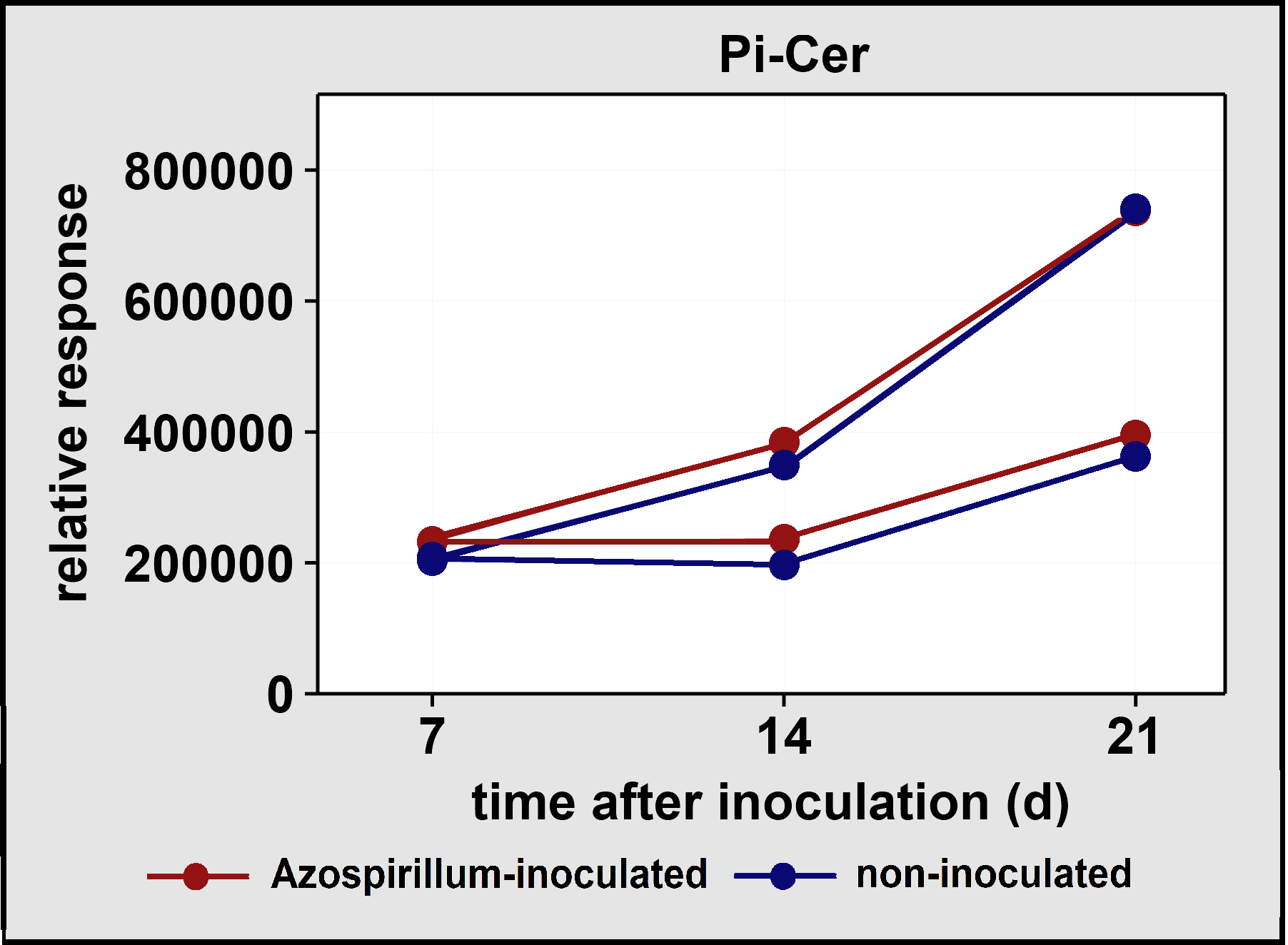

Supplement: Supplementary file 1 [file metabolites-11-00358-s001.zip › Figure S9.tif]
